# Population connectivity in voles (*Microtus* sp.) as a gauge for tall grass prairie restoration in midwestern North America

**DOI:** 10.1371/journal.pone.0260344

**Published:** 2021-12-09

**Authors:** Marlis R. Douglas, Steven M. Mussmann, Tyler K. Chafin, Whitney J. B. Anthonysamy, Mark A. Davis, Matthew P. Mulligan, Robert L. Schooley, Wade Louis, Michael E. Douglas

**Affiliations:** 1 Biological Sciences, University of Arkansas, Fayetteville, Arkansas, United States of America; 2 Southwestern Native Aquatic Resources and Recovery Center, U.S. Fish & Wildlife Service, Dexter, New Mexico, United States of America; 3 Ecology and Evolutionary Biology, University of Colorado, Boulder, Colorado, United States of America; 4 Basic Sciences, St. Louis College of Pharmacy, St. Louis, Missouri, United States of America; 5 Illinois Natural History Survey, University of Illinois, Champaign, Illinois, United States of America; 6 Lincoln Park Zoo, Chicago, Illinois, United States of America; 7 Natural Resources and Environmental Sciences, University of Illinois, Urbana, Illinois, United States of America; 8 Illinois Department of Natural Resources, Gibson City, Illinois, United States of America; University of Nevada, Reno, UNITED STATES

## Abstract

Ecological restoration can promote biodiversity conservation in anthropogenically fragmented habitats, but effectiveness of these management efforts need to be statistically validated to determine ’success.’ One such approach is to gauge the extent of recolonization as a measure of landscape permeability and, in turn, population connectivity. In this context, we estimated dispersal and population connectivity in prairie vole (*Microtus ochrogaster*; N = 231) and meadow vole (*M*. *pennsylvanicus*; N = 83) within five tall-grass prairie restoration sites embedded within the agricultural matrix of midwestern North America. We predicted that vole dispersal would be constrained by the extent of agricultural land surrounding restored habitat patches, spatially isolating vole populations and resulting in significant genetic structure. We first employed genetic assignment tests based on 15 microsatellite DNA loci to validate field-derived species-designations, then tested reclassified samples with multivariate and Bayesian clustering to assay for spatial and temporal genetic structure. Population connectivity was further evaluated by calculating pairwise *F*_ST_, then potential demographic effects explored by computing migration rates, effective population size (*N*_e_), and average relatedness (*r*). Genetic species assignments reclassified 25% of initial field identifications (N = 11 *M*. *ochrogaster*; N = 67 *M*. *pennsylvanicus*). In *M*. *ochrogaster* population connectivity was high across the study area, reflected in little to no spatial or temporal genetic structure. In *M*. *pennsylvanicus* genetic structure was detected, but relatedness estimates identified it as kin-clustering instead, underscoring social behavior among populations rather than spatial isolation as the cause. Estimates of *N*_*e*_ and *r* were stable across years, reflecting high dispersal and demographic resilience. Combined, these metrics suggest the agricultural matrix is highly permeable for voles and does not impede dispersal. High connectivity observed confirms that the restored landscape is productive and permeable for specific management targets such as voles and also demonstrates population genetic assays as a tool to statistically evaluate effectiveness of conservation initiatives.

## Introduction

‘Distribution’ and ‘abundance’ are fundamental natural history aspects of species [1], but the population processes defining these ecological characteristics [2] can be easily disrupted by anthropogenic habitat fragmentation [3]. Most contemporary landscapes reflect large-scale human modifications that constrain species to persist as subdivided, often isolated populations. As a consequence, ‘dispersal’ and thus ‘population connectivity’ have become key ecological parameters that determine long-term population viability [4].

Temperate grassland biomes (>8% of the global landmass [5]) are easily transformed into human settlements and agricultural plots, and species dependent on grassland habitats have been especially impacted. The large-scale agricultural conversion of the Tall Grass Prairie in Midwestern North America [6] emphasizes the extensive human demands imposed upon these biomes. Adoption of row-crop technologies and prolongation of the growing season ([7] Table 1) have substantially enhanced this agricultural capacity [8], but also increased regional greenhouse gas emissions such that they now exceed the national average [9]. In addition, increasing demand for biofuels has further intensified agricultural practices and accelerated the removal of potential ’edge’ habitats along the margins of agricultural fields [10] that previously provided both essential connectivity among habitat fragments for prairie species as well as refugial habitat. Other consequences of large-scale habitat alterations are loss of indigenous biodiversity and increased biotic homogenization [11].

**Table 1:** Vole samples collected during three field seasons (2010–2012) across five SAFE sites in Illinois. Samples were harvested non-invasively from two species, *M*. *ochrogaster* and *M*. *pennsylvanicus*, but only totals are listed. Geographic location of sites in Fig 1.

Site	2010	2011	2012	Totals
Livingston	27	4	10	41
Montgomery	0	56	41	97
Pontiac	21	1	9	31
Prairie Ridge	60	3	32	95
Saybrook	0	19	77	96
Total	108	83	169	360

Habitat restoration can reduce or even reverse the detrimental consequences of these anthropogenic impacts. In the prairie landscape of North America, numerous conservation initiatives have been implemented to improve remnant grassland parcels and increase population numbers of targeted wildlife. Earliest prairie restoration efforts date as far back as the 1930s [12] and now extend from local grass-roots efforts to governmental initiatives such as the State Acres for Wildlife Enhancement Initiative (SAFE; https://www.fsa.usda.gov/programs-and-services/conservation-programs/), a conservation program that aims at converting agricultural land into grasslands by providing annual rental pay to farmers for removing environmentally sensitive areas from production. The goal of SAFE is to promote wildlife through habitat restoration, with the primary target being high priority species designated by U.S. Fish and Wildlife Service as threatened and endangered (T&E), but with other species benefitting as well. The program is voluntary, thus specific locations and numbers of sites may fluctuate over time. In Illinois, SAFE sites are selected in proximity to permanent prairie habitats, with each site comprised of several restoration patches varying in size and time since initial restoration.

However, one critical aspect of restoration is the definition of ’success,’ and which metric(s) can gauge if such a specific goal has been reached. In this study, we evaluated the spatio-temporal genetic structure of two vole species, the prairie vole (*Microtus ochrogaster*) and the meadow vole (*M*. *pennsylvanicus*), as a means of quantifying the effectiveness of prairie restoration efforts within a large-scale agroecosystem. Voles can subsist in small home ranges (100m^2^) within prairie fragments, and are easily captured via live trapping, a technique that yields reliable abundance and movement estimates [13], an important aspect when evaluating effects of fragmentation on population dynamics and demographics [13–15].

The study area encompassed five prairie restoration SAFE sites, isolated within the fragmented row-crop landscape of Illinois and about 30–250 km apart, to test if large geographic distances separating restoration patches in this highly-modified agricultural matrix would act synergistically to curtail vole dispersal [16]. For *M*. *ochrogaster*, relatedness within and among sites was also explored, as it is a highly philopatric and socially monogamous species that often nests cooperatively [17, 18], such that social- and kin-related clusters might impact dispersal. In contrast, *M*. *pennsylvanicus* is promiscuous [19], yet can also form social groups during the non-breeding season [20].

Microsatellite DNA analysis was used to: 1) Quantify genetic diversity in both vole species within and among the five SAFE sites; and 2) Assess levels of temporal and spatial gene flow among habitat patches. Population genetic data reflect dispersal of organisms through complex environments, and hence can reveal if connectivity indeed exists amongst habitat patches widely-separated within an anthropogenically-modified landscape [21]. This approach also provides an estimate of landscape permeability, an important component of restoration programs within agroecosystems where potential habitat rehabilitation is often restricted to isolated patches, but where connectivity can be established through edge habitat within surrounding areas [22]. Our expectation was that limited connectivity among study sites would be reflected in strong population structure, particularly given the life histories of the study species, and the relative isolation of restoration patches within the agroecosystem.

## Materials and methods

### Sampling and DNA extraction

**Fig 1:**
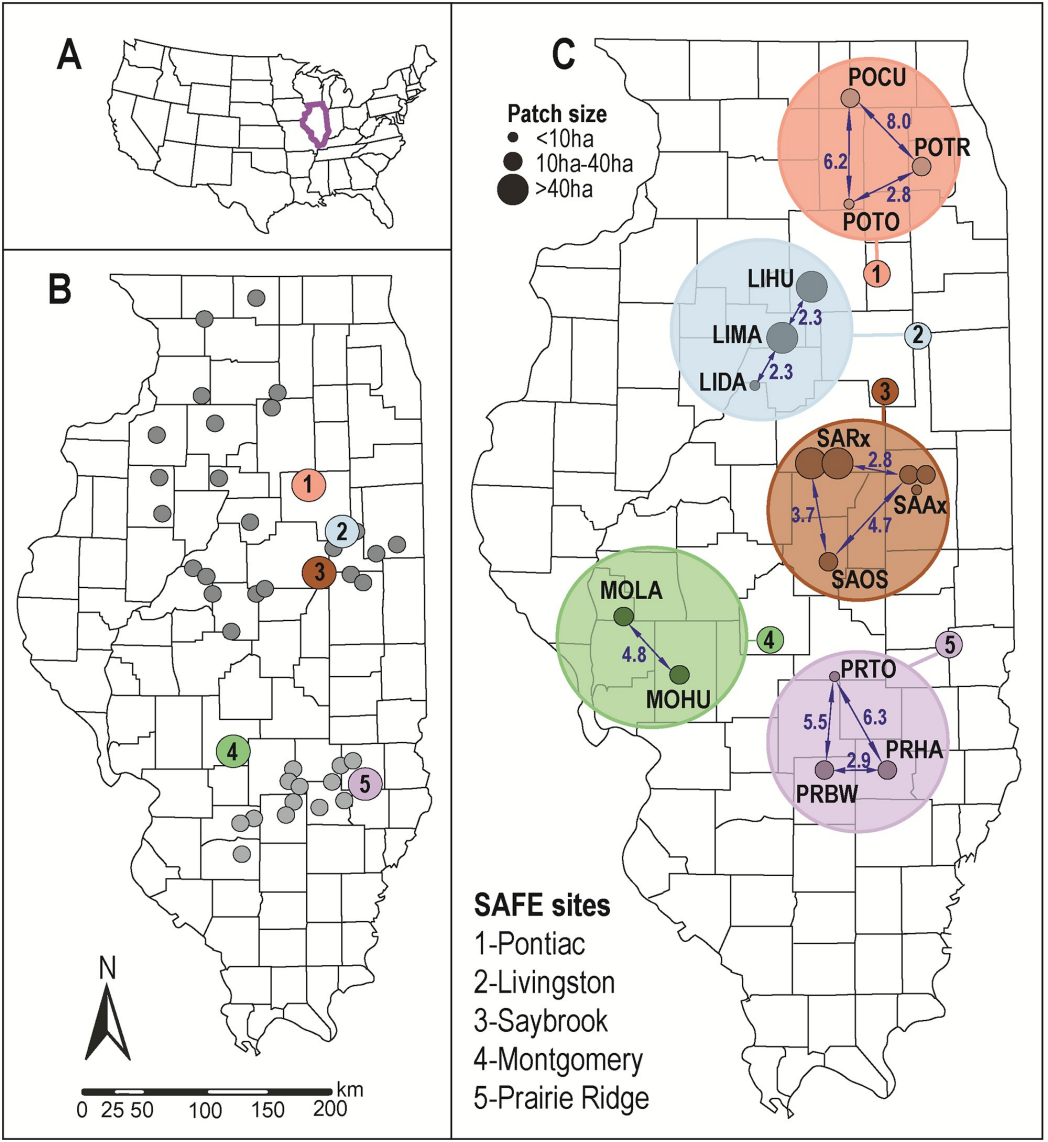
Map depicting State Acres for Wildlife Enhancement (SAFE) restoration sites in Illinois. (A) Location of Illinois (purple) in the USA. (B) Distribution of SAFE sites (circles) in Illinois: color = sampled sites; dark grey sites in the Grand Prairie region; light gray = sites in the Southern Till Plain. (C) Schematic distribution of restoration patches (small circles) at sampled sites (large circles): arrows = inter-patch distances (in km); circle size = patch area (in ha). The map was generated in ArcGIS 10.4.1 using publicly available data downloaded from government institutions, with boundaries derived from Topologically Integrated Geographic Encoding and Referencing (TIGER) data via U.S. Census Bureau. Additional details on patches provided in S1 Table.

Voles were live-trapped from mid-May through mid-August (2010–2012) as part of a concurrent study [23] at representative SAFE sites that evaluated response of small mammal communities to prairie restoration efforts. Tissue samples were collected from five SAFE sites approximately 35–250 km apart (Fig 1B) in central/ south-central Illinois. Each site contained a mosaic of restoration patches (Fig 1C) varying in area (<2ha to >65ha), distance from other patches (0m to 8km), or time since initial restoration (1 year to >10 years) (S1 Table). The surrounding landscape was dominated by row crop agriculture, but restored patches were connected to various degrees by fencerows, roadside ditches, or grass waterways. Restoration was conducted by seeding previous cropland with either CP1 (cool-season grasses and legumes), CP2 (warm-season grasses), or mixtures [24]. However, neither patch size nor seeding type impacted vole abundance [23], but population cycles were seemingly an overriding factor. Sampling was conducted via six transects of 15 traps each set 7m apart within each restoration patch, with coverage defined by patch size. Trapping occurred over three consecutive evenings (N = 90 traps/ patch/ night; [24]), and was approved by the Institutional Animal Care and Use Committee (IACUC), University of Illinois/Urbana-Champaign.

Ear tissue was sampled from each captured vole, with a maximum of 30 samples/ patch/ year and stored in 95% EtOH at -20°C for subsequent genetic analyses. Whole genomic DNA was extracted using Promega Wizard Kit (2010–2011) or Qiagen DNeasy Blood and Tissue Kit (2012) and quantified using an Implen Pearl P-300 nanophotometer.

### Microsatellite DNA genotyping

Microsatellite loci previously developed for vole species were evaluated for consistent cross-amplification in *M*. *ochrogaster* and *M*. *pennsylvanicus*. A set of 23 loci was selected for genotyping and combined into six multiplex panels using fluorescently labeled forward primers (S2 Table). Amplifications were conducted in 10–15μl volume polymerase chain reactions (PCR) using approximately 10–15ng template and following standard procedures. Additional details on reaction conditions and cycling profiles are provided in S2 Table.

#### Fragment analysis

Microsatellite DNA fragments were resolved on an automated Applied Biosystems (ABI) Prism 3730xl GeneAnalyzer at the W. M. Keck Center, University of Illinois, Champaign. An internal size standard (Liz 500) was run with each sample, and alleles were scored using GeneMapper© 4.1 software (ABI). Genotypes were partitioned by species, site, and sampling period, then tested via Micro-Checker 2.2.3 [25] for null alleles, large allele dropout and scoring errors. All pairs of loci were tested for linkage disequilibrium (Markov Chain parameters: 10,000 dememorization steps, 500 batches, 5,000 iterations), and each locus evaluated for deviations from Hardy-Weinberg equilibrium (HWE) using exact tests (GenePop 4.0; [26]) with Bonferroni correction for multiple comparisons [27].

### Assignment of individuals to species

The two vole species are morphologically similar and accurate identification can be difficult, particularly when non-lethal sampling is conducted. To verify field-based species identification, we employed the population assignment option in GenAlEx 6.50 [28] to cluster samples according to similarities in genotype. Genetic-based species assignments were then used to re-classify individuals to species and re-evaluate distinct gene pools using Bayesian clustering (Structure 2.3.4. [29]); with admixture ancestry and correlated allele frequency options selected [30].

#### Molecular taxonomy

To further verify species designation, we genotyped a diagnostic locus (*Microtus avpr1a* gene [31]) that identifies *M*. *ochrogaster* with an allele of ~600–800bp, and *M*. *pennsylvanicus* with one of ~200–300bp. Primers and PCR protocols were adapted from previous studies to test a subset of samples (N = 68) for concordance between field- and genetic-based species identification [32–34], and diagnostic alleles visualized by separating PCR products on 2.5% agarose gel stained with GelGreen (Biotium Inc., Hayward, CA) and examined on a bluelight transluminator.

### Genetic diversity and structure of vole populations

Standard population genetic parameters were calculated to quantitative diversity within and among sites. These included measures of allele frequencies and heterozygosity and were estimated for each species at each site using GenAlEx. Values for allelic richness (*A*_R_) and private allelic richness (*P*_AR_) were derived from rarefaction and corrected for sample sizes (*N* = 23; HP-Rare v. June-6-2006; [35]). Pairwise relatedness (*r*) among individuals was calculated using the Ritland (1996) estimator in GenAlEx to reduce potential bias in genetic analyses by inadvertent comparison of related individuals captured in localized trapping transects (a particular concern when evaluating spatial genetic structure). To mitigate, we subsequently removed individuals at random from pairwise comparisons when *r* ≥ 0.25.

#### Multivariate clustering

To visualize genetic structure, we first performed a principal component analysis (PCA) using the *dudi*.*pca* function in the R package Adegenet [36]. Because PCA captures both among-group (i.e., ‘population structure’) and within-group genetic variation [37], a discriminant analysis of principal components (DAPC) was subsequently performed to isolate the among-group components [36]. An automated cross-validation procedure was used to select the optimal number of PCs by iteratively exploring classification error as a function of the number of retained PCs. Here, 80% of the samples served as a ‘training set’ for the remaining 20% of samples. All evaluations utilized the *xvalDapc* function of Adegenet [36], as implemented in a custom R script (*vol_dapc*.*R* in the Open Science Repository; doi: 10.17605/OSF.IO/K2M8W).

#### Bayesian clustering

To further assess distribution of gene pools within and among sampling locations assignment tests were conducted (Structure; [29]).Ten replicates were run for *K*-values ranging from 1–10 using a burn-in of 500,000 iterations, followed by 1,000,000 Markov Chain Monte Carlo (MCMC) replicates. To account for potential multimodality per *K*, results were processed in Clumpak [38]. The optimal number of clusters for each simulation (per Clumpak) was evaluated by the ad hoc statistic ‘Δ*K*’ [39], and also the probability by *K* [29]. This was done because Δ*K* is unable to evaluate validity of *K* = 1, and also has a propensity to select *K* = 2 [39, 40].

Genetic structure detected within localities for *M*. *pennsylvanicus* suggested the potential for aggregation of related individuals (i.e., kin structure). Therefore, family structure was evaluated (Colony v2.0.6.6; [41]) to determine if clusters corresponded with family groups. Analytical methods allowed for female polygamy as well as inbreeding and program parameters were selected as ’very long’ run length, full-likelihood analysis and ’very high’ precision.

#### Power analysis

To measure our ability to resolve population structure, a power analysis was conducted (PowSim; [42]) utilizing both available test options (i.e., χ2 and Fisher exact tests). The Fisher tests were performed using 1,000 dememorization steps followed by 1,000 iterations in 100 batches. Empirical allele frequencies, sample sizes, and number of populations were evaluated, while an initial effective population size of 500 was assumed. The time of divergence required to detect population differentiation was estimated by varying number of generations impacted by genetic drift (*t*) from 2–20. Power was assessed as the number of significant tests observed per 10,000 replicates.

#### Pairwise *F*_ST_

Population connectivity was evaluated using pairwise *F*_ST_, a standardized index that reflects average gene flow over time, with spatial/ temporal patterns estimated within years among sites, and among years within sites (GenAlEx, with 9,999 permutations and missing data interpolated). Gene flow could only be reliably assessed for *M*. *ochrogaster*, due to variability in numbers of samples obtained at sites among years for *M*. *pennsylvanicus*, as well as the uneven distribution of samples following genetic re-classification of samples.

To assay spatial connectivity, we derived pairwise *F*_ST_ values for *M*. *ochrogaster* at three sites in 2010 (i.e., Livingston, Pontiac, and Prairie Ridge), and between two sites in 2012 (i.e., Montgomery and Prairie Ridge). To test for potential impacts of environmental perturbations (e.g., drought in 2012; [21, 24]), we also calculated pairwise *F*_ST_ estimates between years at two sites (Montgomery: 2011 *versus* 2012; Prairie Ridge: 2010 *versus* 2012). Sample sizes were too small at other sites or time intervals to conduct either spatial or temporal comparisons.

### Estimates of migration and effective population size

Individual movements can influence population dynamics, and two are of particular importance in this regard: Colonization (i.e., movement into an unoccupied habitat patch) and migration (i.e., immigration into occupied patches). For example, the annual replanting of crops is an agroecosystem dynamic that can alternately convert vole populations into sinks that must be potentially colonized from a source [43].

#### Recent migration among SAFE sites

Potential F1 descendants of migrant individuals were identified using GeneClass2 2.0; [44]) and by selecting the ’L_home’ test statistic (i.e., likelihood of obtaining the genotype of an individual from the sampled population under the assumption that not all source populations were sampled). A Monte Carlo resampling method was selected, with 10,000 bootstraps and a threshold value of 0.01, as it improves performance when identifying first generation migrants while also controlling for Type-I error rates [45].

#### Effective population size (*N*_e_)

Trapping success was uneven among years, impacted by an apparent regional population decline in 2011 and a substantial drought in 2012 [23, 24]. To gauge the effects of these perturbations on vole populations, we quantified *N*_e_ (LDNE; [46]) based upon estimates of linkage disequilibrium (i.e., a non-random association of independent alleles with haplotypes occurring in unexpected frequencies; [47]). *N*_e_ reflects the rate of genetic drift (i.e., random fluctuations in allele frequencies over time; [13]), as well as the effectiveness of selection and migration. It also indicates heterozygosity loss and links strongly with demographic factors such as sex ratio, population size, and lifetime fitness.

*N*_*e*_ and associated 95% jackknife confidence intervals were calculated using NeEstimator v2.1 [48], with rare alleles (*P*_CRIT_) excluded from analysis [49] (i.e., *P*_CRIT_ = 0.02 when *N* ≥ 25). Due to sample size limitations, *N*_e_ could only be calculated for *M*. *ochrogaster* at Prairie Ridge (2010 *versus* 2012) and Montgomery (2011 *versus* 2012). Mean *N*_*e*_ at these two sites were compared per year by implementing Welch’s t-test for unequal variances. This approach is more robust than Student’s *t*-test and maintains Type-I error rates despite inequalities among variances and sample sizes. The test was performed in R [50] using summary statistics and a suitably-modified web-based program: (http://stats.stackexchange.com/questions/30394/how-to-perform-two-sample-t-tests-in-r-by-inputting-sample-statistics-rather-tha).

#### Isolation-By-Distance (IBD)

Finally, we evaluated the degree to which dispersal limitation has contributed to spatial genetic structuring within the two species by calculating genetic differentiation as a function of geographic distance between sample sites. We first calculated pairwise genetic distances among localities (i.e., Edwards distance; [51]) using the *dist*.*genpop* function in Adegenet [36]. We then used the Mantel test to compare these data against with pairwise geographic distances derived from latitude and longitude coordinates (S1 Table), with significance computed using 999 random permutations [52].

## Results

### Sampling, genotyping, and species assignments

A total of 360 voles were trapped across five prairie restoration sites over three field seasons (Table 1). Of these, 194 were field-identified as *M*. *ochrogaster*, and 166 as *M*. *pennsylvanicus*. All samples were genotyped across 23 microsatellite loci, with eight subsequently removed due either to null alleles or scoring issues, leaving 15 loci for data analyses. In addition, 46 samples missing data at more than one locus were excluded, leaving 314 individual genotypes for evaluation (S1 Table). Of these, 271 had complete genotypes and 43 lacked alleles at a single locus. Linkage disequilibrium was detected but was inconsistent across temporal periods or sites, and thus attributed to demographic effects on genetic structure rather than non-independence amongst loci.

Genotype-derived species assignments were concordant with 75% of field identifications based on morphology (236 of 314). Among the remaining 78 field identifications, 11 *M*. *ochrogaster* and 67 *M*. *pennsylvanicus* were genetically reclassified (S1 Fig), resulting in 231 *M*. *ochrogaster* and 83 *M*. *pennsylvanicus* genotypes, respectively (S1 Table). A subsequent Bayesian cluster analysis using reclassified species identifications consistently allocated all 314 samples (S1 Fig). Screening with the diagnostic *avpr1a* locus confirmed species-level classifications for 97% of test samples (65/67), with two individuals (0.6% of 314) identified as hybrids, suggesting a rare occurrence of interbreeding between the two study species. The Bayesian assignment plot (S1 Fig) also reflected some individuals with admixed ancestry, including the two individuals of hybrid origin. Genetic species assignments rather than field identification were employed in subsequent analyses (S1 Table).

### Genetic diversity and population structure

Microsatellite DNA polymorphism was high in both species, as indicated by mean numbers of alleles (*N*_A_) and observed heterozygosity (*H*_o_) for *M*. *ochrogaster* (N_A_ = 22.7; *H*_o_ = 0.80) and *M*. *pennsylvanicus* (*N*_A_ = 16.3; *H*_o_ = 0.70), respectively (Table 2). Pairwise *F*_ST_ values by patch and site were non-significant for *M*. *ochrogaster* at both spatial and temporal scales, save for comparisons involving a patch in the southernmost SAFE site (i.e., Tombstone patch at Prairie Ridge (S3 Table).

**Table 2:** Genetic diversity in 212 *M*. *ochrogaster* (MIOC) and 75 *M*. *pennsylvanicus* (MIPE) based on 15 microsatellite loci.

	Site	*N*	*N*_A_	*N*_A_ SE	*A*_R_	*A*_R_ SE	*P*_AR_	*P*_AR_ SE	*H*_o_	*H*_*o*_ SE
MIOC	Livingston	28	13.5	1.4	12.4	1.2	1.4	0.3	0.81	0.04
	Montgomery	71	17.7	1.8	12.7	1.1	1.6	0.3	0.79	0.04
	Pontiac	23	13.2	1.1	13.0	1.1	1.4	0.3	0.83	0.04
	Prairie Ridge	85	16.2	1.7	12.1	1.1	1.0	0.3	0.80	0.05
	Saybrook	5	6.3	0.5	-	-	-	-	0.84	0.06
MIPE	Livingston	6	6.3	0.5	-	-	-	-	0.63	0.07
	Saybrook	69	15.7	1.3	12.0	0.9	7.15	0.7	0.70	0.06

**S**amples were collected from five SAFE sites. *N* = number of individuals genotyped; *N*_a_ = mean number of alleles per locus; *N*_a_ SE = standard error *N*_a_; *A*_R_ = allelic richness corrected for sample size; *A*_R_ SE = standard error *A*_R_; *P*_AR_ = private allelic richness corrected for sample size; *P*_AR_ SE = standard error *P*_AR_; *H*_o_ = observed heterozygosity; *H*_o_ SE = standard error *H*_o_.

**Fig 2:**
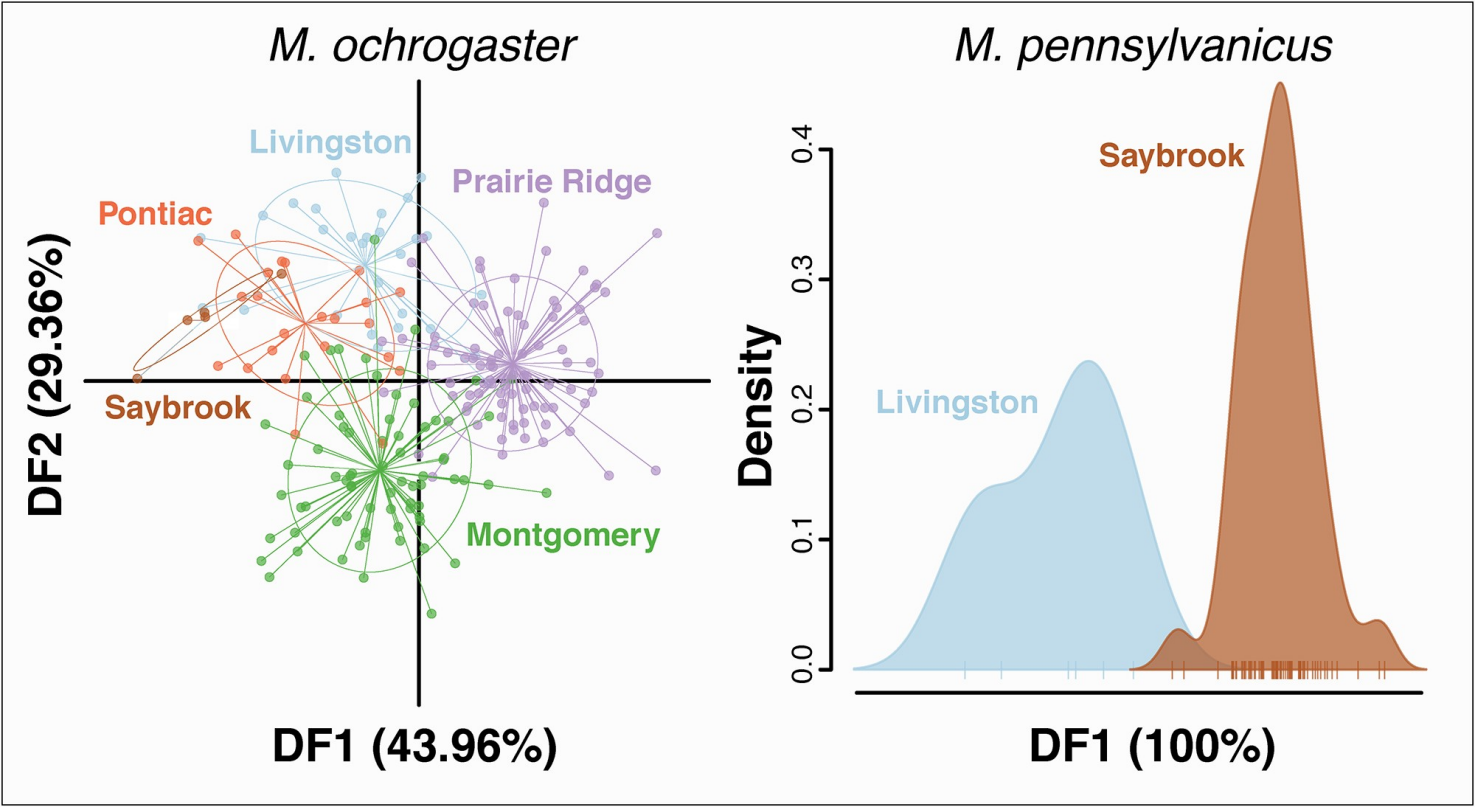
Discriminant analyses of principal components (DAPC) for *M*. *ochrogaster* and *M*. *pennsylvanicus*. Genetic clusters found at five SAFE sites: Livingston, Montgomery, Pontiac, Prairie Ridge, and Saybrook. Data derived from 15 microsatellite loci. Colors reflect unique sites and are consistent across species; individuals are represented as points for *M*. *ochrogaster* and ticks for *M*. *pennsylvanicus*. The percentage of variation from the discriminant analysis captured by each discriminant function axis (DF) is in parentheses.

PCA revealed a greater spread within rather than among sites for both vole species, with the first two PC axes capturing 3.76% and 3.29% of variation in *M*. *ochrogaster*, and 6.12% and 4.96% of variation in *M*. *pennsylvanicus* (S2 Fig). For the DAPC (Fig 2), 60 PCs were retained for *M*. *ochrogaster*, and 30 for *M*. *pennsylvanicus* (S3 Fig). Genetic clusters in the DAPC overlapped for *M*. *ochrogaster*, but with spatial relationships generally mirroring geography, showing a slightly stronger association between the three norther-central SAFE sites (Pontiac, Livingston and Saybrook) and the two southern sites (Montgomery and Prairie Ridge), respectively (Fig 2). Clusters for *M*. *pennsylvanicus* overlapped less so, although samples were only available for two sites (i.e., Livingston and Saybrook; Fig 2).

**Fig 3:**
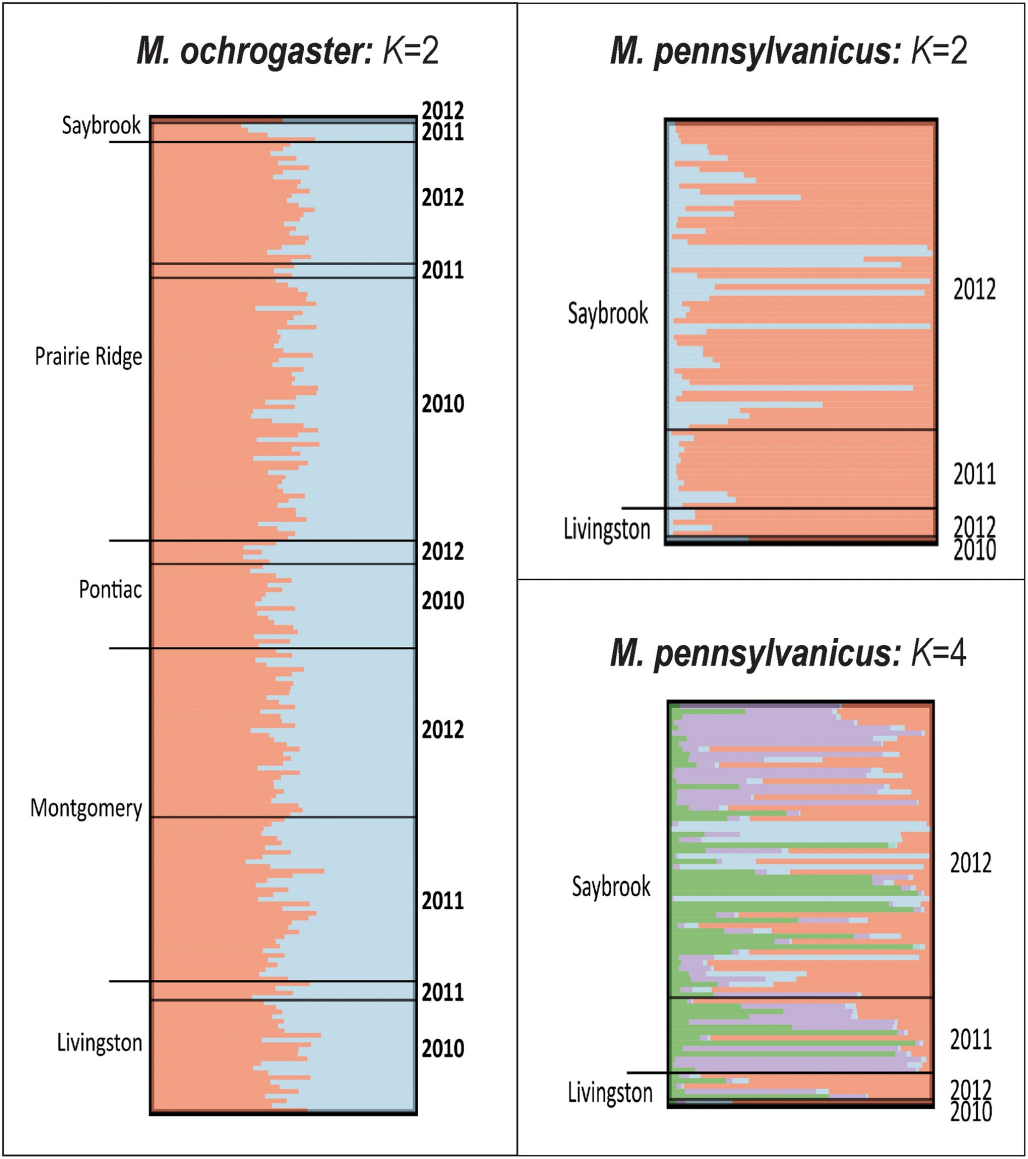
Spatial and temporal genetic patterns in *M*. *ochrogaster* and *M*. *pennsylvanicus*.

Assignment tests failed to recover distinct gene pools for *M*. *ochrogaster*, despite the selection of *K* = 2 (Fig 3) by the Δ*K* statistic (S4 Fig). Rather, the species was represented as a single homogenous population across the study area, in concordance with the selection of *K* = 1 (Pr(*K*) statistic) as the best explanation of population structure (S4 Fig). In contrast, *M*. *pennsylvanicus* (Fig 3) was represented by either two clusters (ΔK; S4 Fig) or four [Pr(*K*) S4 Fig]. However, one cluster in both *K* = 2 and *K* = 4 mostly corresponded to a family group recovered by Colony. Here, four (of 12) individuals (33%) in this family group were assigned fractionally to the cluster by Structure (ancestry < 0.15). We interpreted this as a consequence of the relatively low probability assigned to the family group by Colony [*P*r = 0.7038; a list of family groups is provided in the Open Science Framework repository (doi:10.17605/OSF.IO/K2M8W)]. No other population or family structure was concordant among both Structure and Colony outputs. Scant evidence of spatial or temporal structure was apparent for either species.

Power analysis indicated adequate power to discern population clusters in both species (S5 Fig). This was true across all temporal scales in *M*. *ochrogaster*, despite an apparent lack of structure in the empirical data. However, *M*. *pennsylvanicus* shows sensitivity to divergence time in that a minimum of 15 simulated generations was required for significant population differences in >90% of replicates. This is likely a consequence of low sample size (N = 6) associated with the Livingston site.

Plots are based on Bayesian clustering (Structure v2.3.4). Colors reflect distinct gene pools and horizontal bars within plots represent individuals. The proportion of each color within an individual bar reflects the probability of ancestry. Samples are grouped by site and year (separated by black bars). Sample sizes are in Table 2.

### Relatedness

We removed 19 individuals from our analyses because of relatedness (*r)* values >0.25. Average within-patch *r*-values ranged from 0.009–0.106, with higher values generally seen among individuals at the southernmost SAFE site (Prairie Ridge; S4 Table), where relatedness at one restoration patch (PRHA) differed significantly between 2010 and 2012 (*t* = -3.06, *P*<0.028), whereas those for the other patch (PRTO) did not (*t* = -0.54, *P*>0.6). An insufficient sample size prevented such a comparison for the third patch.

Average pairwise relatedness and geographic distance were significantly but inversely related across patches and sites in 2010, with relatedness diminishing as distances increased (3 sites, 6 patches; *P* = 0.027). However, relatedness *versus* distance was non-significant in 2012 (2 sites, 4 patches; *P* = 0.13).

### Estimates of migration and effective population size

Estimates of migration (GeneClass2) indicated contemporary movements of individuals among SAFE sites (Table 3). In *M*. *ochrogaster*, five individuals (= 2%) were identified as potential migrants based on an assignment probability threshold <0.01. No potential migrants were identified for *M*. *pennsylvanicus*, but it was only captured at two patches, with only six individuals sampled from Livingston and the remaining 77 from Saybrook.

**Table 3:** Rates of migration *versus* residency for *M*. *ochrogaster*.

**2010**	**Livingston**	**Prairie Ridge**
Livingston	**0.767 (0.418)**	0.291 (0.066)
Prairie Ridge	0.233 (0.216)	**0.709 (0.485)**
**2012**	**Montgomery**	**Prairie Ridge**
Montgomery	**0.858 (0.51)**	0.265 (0.003)
Prairie Ridge	0.142 (0.0003)	**0.735 (0.341)**

Estimates were derived within and between SAFE sites for years and sites with sufficient sample sizes: 2010 (Livingston *N* = 24; Prairie Ridge *N* = 57) and 2012 {Montgomery *N* = 35; Prairie Ridge *N* = 30). Values are posterior estimates for mean and 95% highest posterior density (in parentheses). Bold values = residency estimates.

**Fig 4:**
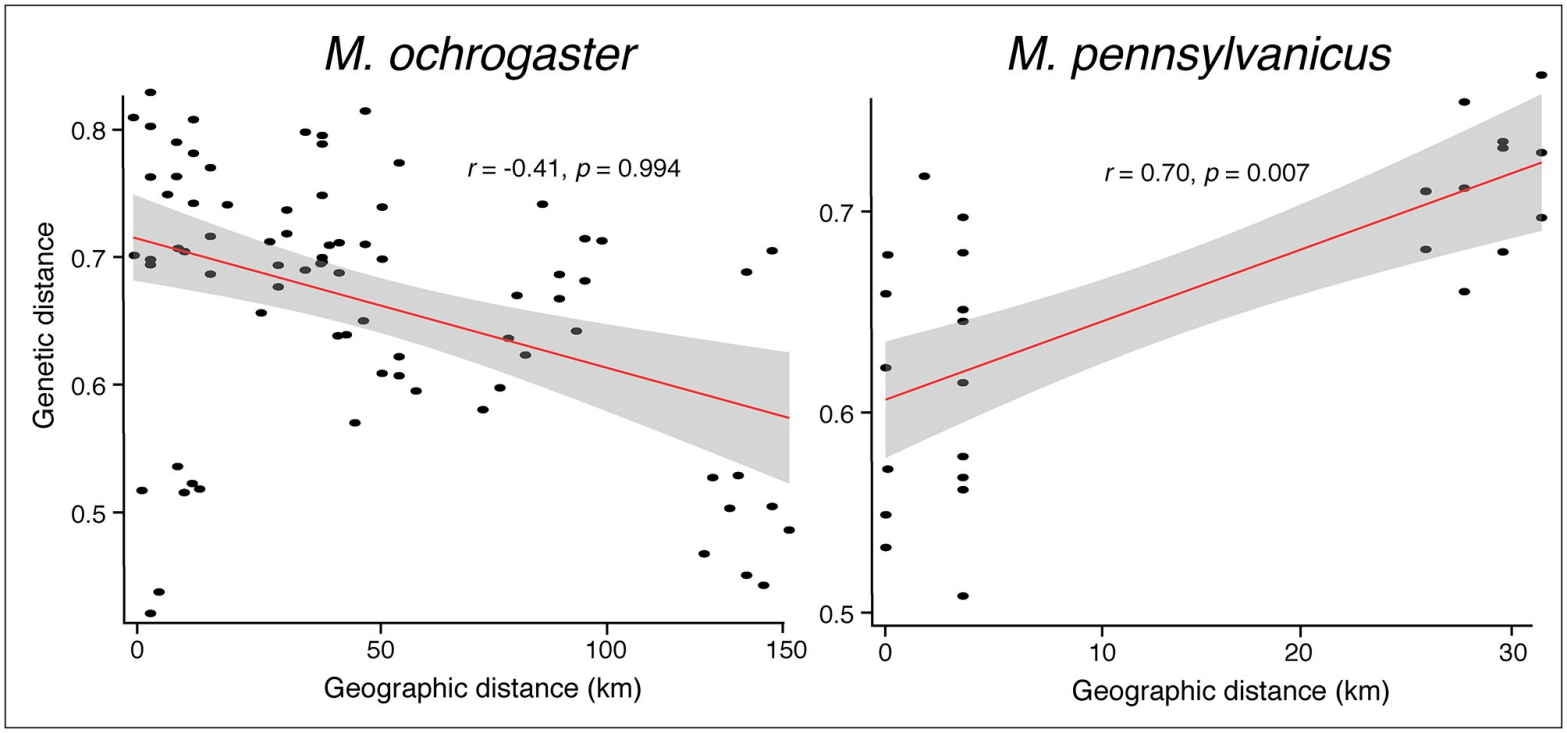
Patterns of isolation-by-distance among *M*. *ochrogaster* and *M*. *pennsylvanicus*. For *M*. *ochrogaster* estimates are based on samples from five SAFE sites (= Livingston, Montgomery, Pontiac, Prairie Ridge, and Saybrook) and for *M*. *pennsylvanicus* on two sites (= Livingston and Saybrook). The X-axis represents pairwise geographic distances computed from latitude and longitude coordinates, whereas the Y-axis represents genetic distances computed among localities using allele frequencies for 15 microsatellite loci. Lines represent linear models, with confidence intervals in gray. Numbers above linear regression lines represent Mantel *r* and *p*-values estimated from 999 random permutations.

Tests for IBD notably differed between species, in a manner concordant with the migration analysis. Here, *M*. *ochrogaster* showed little evidence for dispersal limitation over the distances examined (maximum pairwise distance = 143km), with a negative but non-significant relationship between geographic and genetic distances (Mantel *r* = -0.41; *p* = 0.994; Fig 4). However, results for *M*. *pennsylvanicus* reflected an IBD pattern (Mantel *r* = 0.70; *p* = 0.007), despite a much smaller maximum observed distance (= 31.6km; Fig 4).

#### Population fluctuations

In *M*. *ochrogaster N*_e_ declined (but non-significantly) at Prairie Ridge from 148 in 2010 (95% CI = 83–479) to 58 in 2012 (95% CI = 32–197; *t* = 0.82; *P*<0.4136). At Montgomery, *M*. *ochrogaster* populations also displayed a reduced *N*_e_ estimate, with values declining from 579 (95% CI = 67—∞) in 2011 to 169 (95% CI = 67—∞) in 2012 (*t* = 3.06; *P*<0.0032). A test of significance could not be performed due to confidence intervals trending to infinity. Comparison of each confidence interval demonstrated a lack of significance, as they overlapped reciprocally with *N*_e_ estimates for each year (S6 Fig).

## Discussion

Ecological restoration is an integral aspect of biodiversity conservation and wildlife management in anthropogenically fragmented habitats, but it must be quantitatively assayed to determine if ongoing efforts are indeed effective. Thus, metrics are needed to quantify how extensive and at what level restored patches are colonized and interconnected [53]. Habitat fragmentation is often best assessed by estimating connectivity among populations of small mammals [54], where level of isolation (i.e., reduced gene flow) may be ecologically more relevant than patch size, particularly when agricultural plots are converted into natural habitat [55]. However, population connectivity is less well studied within large, homogenized agricultural landscapes when compared to natural habitats, due largely to the lack of diversity in small mammal communities [56]. In addition, those studies that evaluated genetic structuring and diversity in agroecosystems reported conflicting results [57].

Our empirical evaluation of genetic structure and population connectivity in two vole species provides insights into the mechanisms by which restored patches of an agroecosystem may facilitate population persistence in target species. A lack of spatial genetic structure in our data, even over larger geographic distances, suggests the landscape mosaic dominating Illinois is highly permeable for voles and does not limit their dispersal. Despite extensive fragmentation, restored patches are sufficiently connected via edge habitat that provides corridors [58], allowing voles to rapidly colonize and supporting demographic resilience [59, 60]. Thus, our results help sustain restoration goals while documenting the effectiveness of management options.

### Taxonomic uncertainty and species abundances

The correct identification of species in the field is a fundamental assumption when ecological hypotheses are tested, and when conservation and management initiatives are implemented. Species uncertainty, particularly among phenotypically-similar sibling species, can bias population estimates [61]. *Microtus* spp., including the two vole species in this study, are difficult to distinguish in the field, with a diagnosis often based on qualitative characters that relate to pelage, as well as differences in tooth morphology [62].

Fortunately, species-diagnostic DNA markers can unambiguously discriminate among sympatric species [63], as well as decipher potential hybridization and introgression. In our data 25% of field identifications did not match those derived genetically for *M*. *ochrogaster* and *M*. *pennsylvanicus*. Similar disparities have been observed in other studies [31].

Hybridization and resulting admixture represent another confounding factor when taxa are identified based on phenotype [64]. In our study, genotypes of two samples reflected admixture, and both were from a SAFE site (Saybrook) with a preponderance of *M*. *pennsylvanicus*. Admixture between species is often promoted by range shifts among distributions previously discrete. The construction of Illinois interstate highways have had variable impacts on wildlife [65], and seemingly facilitated a range expansion of *M*. *pennsylvanicus* from northern into central Illinois during the 1970’s. This created a local contact zone between the two species [66] and, in turn, may have facilitated opportunities for inter-species mating. These aspects underscore the necessity of molecular species identification, particularly for sympatric voles.

Field studies showed that the two vole species responded differently to the environment, particularly as it related to trophic and habitat preferences [67, 68]. While *M*. *ochrogaster* was more tolerant of sparse cover, *M*. *pennsylvanicus* preferred more dense vegetation. Habitat preference may thus explain the disparity in our sample sizes, particularly given that vegetation communities varied among study sites [23, 24]. Despite efforts to obtain equal numbers for each species, our samples were heavily skewed towards *M*. *ochrogaster*, particularly after genetically reclassifying 67 samples initially field-identified as *M*. *pennsylvanicus* (S1 Fig).

Voles are important components of grassland ecosystems [69] and they respond differentially to vegetation management. Thus, accurate identification is clearly important when the success of prairie restoration is being evaluated, and our study documents the effectiveness of genetic data in accurately diagnosing species. Species-specific assays, such as the *Microtus avpr1a* gene applied in this study, make molecular taxonomy easy and affordable. In addition, as demonstrated herein, appropriate analytical approaches such as Bayesian clustering can also accurately diagnose species if indeed population genetic data are available.

### Dispersal capacity in agricultural landscapes

Spatially-structured habitats such as the agroecosystems dominating the midwestern plains of North America are characterized by heterogeneity in patch quality, and this serves to modulate demographic processes such as population density and dispersal rates [13]. Small mammals represent a substantial component of the biodiversity within grassland ecosystems, with deer mice (*Peromyscus* sp.) and vole (*Microtus* sp.) being both ubiquitous and linked to complex trophic interactions [69]. However, these dynamics are strongly influenced in modified agricultural landscapes by crop type, farming practices, and vegetational structure [68].

Experimental manipulations of patch quality demonstrated that food availability and predation risk indeed affected dispersal in *M*. *ochrogaster* [70], as well as intrinsic population demographics (e.g., growth rates and reproductive success; [71]), but not social structure [70, 72]. Habitat fragmentation also impacts movements, and a 7-year mark-recapture study of *M*. *ochrogaster* [15] showed less frequent dispersal occurred when habitat fragmentation was elevated, but distances so moved were greater. This frequency *versus* distance was interpreted as a balance between costs associated with habitat loss *versus* higher predation risk via distances traveled.

Given these observations, we predicted that the highly fragmented and agriculturally dominant landscape of central Illinois would impede vole dispersal, and the cumulative effects of limited gene flow would manifest themselves in genetic structure at larger spatial scales [59]. However, for *M*. *ochrogaster* we detected neither significant structure (Figs 2 and 3), nor genetic divergence among sites (S3 Table), which suggests the maintenance of connectivity across the study area. Here, the many intervening, but unsampled SAFE sites (Fig 1B) may have facilitated dispersal and persistence as a stepping-stone model with edge-habitat serving as corridors.

‘Border habitats’ such as fencerows, roadside ditches, and waterways can also promote the recolonization of restored grasslands [73], and may be especially important in maintaining vole populations [60], particularly given the rapid demographic response often observed after a population crash [74]. In this sense, restored grasslands can serve as a ‘source,’ buffering those population declines induced by the annual harvesting/ replanting of crops [23, 43]. In the context of vole population cycles, this would also promote demographic resilience [75, 76]. The spatial clustering we detected in *M*. *pennsylvanicus* is most likely related to social behavior (see below).

### Genetic structure in voles

Dispersal in voles is modulated spatially and temporally [43] by landscape characteristics, population fluctuations, and social systems. Thus, gene flow is impacted by both intrinsic (life history) and extrinsic (environment) processes, and population genetic analyses can reveal which of these forces may be at play.

#### Structure and spatial scale

Geographic distance can be a primary factor in determining the dispersal of rodents in converted ecosystems [77]. However, we failed to detect significant spatial structure or patterns of IBD in *M*. *ochrogaster* (Fig 4). Lack of genetic structure could be due to insufficient resolution of the markers used (false negative or type II error), but our power analyses demonstrated that genetic diversity, at least in *M*. *ochrogaster*, was sufficient to detect structure, if present (S5 Fig). High levels of connectivity were also reflected in low, mostly non-significant *F*_ST_ values (S3 Table), as well as potential migrants between sites (Table 3). *Microtus pennsylvanicus* was only available from two SAFE sites, and the significant IBD pattern (Fig 4) was likely due to clustering of related individuals.

#### Structure and demographic cycles

Fluctuations in population sizes and densities among years can also elicit temporal shifts in genetic structure [43, 73]. Both vole species display multi-annual (but non-synchronous) population density cycles [67, 74]. Higher densities lead to increased dispersal tendencies and thus gene flow, whereas low densities reduce both, thus increasing potential population divergence via genetic drift [43]. We did not detect significant temporal shifts in population genetic parameters, with estimates of *N*_e_ and relatedness stable over time (S6 Fig).

Our sampling results were uneven among years, impacted by an apparent regional population decline (2011) and a substantial drought (2012). Most individuals (47%) were collected in 2012, fewer in 2010 (30%), and least in 2011 (23%), with but a few samples (N<5) available for three sites in 2011, and none at two sites in 2010 (Table 1). Population fluctuations occurred among years, per CPUE estimates (Catch-per-unit-effort; [23]), likely reflecting variance in population densities. However, densities may not have been similar across larger restoration patches, and trapping may have occurred in high- or low-density areas within a given patch. Genetic data are useful to infer population connectivity at large spatial scales, but they cannot inform about demographic connectivity unless direct measures of relatedness are estimated [78].

#### Structure and social systems

At more local scales, genetic structure in voles can be attributed to spatial clustering of kin [18], or reflect sex-specific differences in habitat use [73]. The two vole species differ in this regard, with *M*. *ochrogaster* socially monogamous with strong pair bonds and an elevated degree of philopatry and sociality [79]. Preferences for familiar peers are maintained in part by aggression toward unfamiliar individuals, as in mate partnerships [80]. Conversely, *M*. *pennsylvanicus* is promiscuous [19] and males are more likely to disperse [70], with reduced aggression toward unfamiliar conspecifics [80], and formation of social groups in the non-breeding season [20] being facilitated.

Therefore, both species seasonally gather in communal groups, and clustering of *M*. *pennsylvanicus* in our data is attributed to the aggregation of related individuals (Fig 3). Clustering of kin could also contribute to our observation of temporal stability in genetic diversity. A study involving the root vole, *M*. *oeconomus*, is consistent with this hypothesis, in that temporal stability of genetic composition was attributed in part to individuals immigrating from nearby areas that were closely-related genetically [72].

### Restoration success depends upon the goal

Both patch size and connectivity (effective corridors) are essential to sustain genetically diverse populations [81], and thus underscore the success of ecological restoration. In our study, population connectivity among restoration sites was high and sustained over large geographic distances. Voles seemingly disperse through this landscape mosaic and can quickly colonize newly-restored patches, and even smaller patches appear suitable to sustain populations if indeed connectivity is maintained [23]. Thus, the SAFE model seemingly achieves restoration goals for those small mammals with early colonizing ability.

Medium- to large-sized species are more sensitive to patch size and landscape context in the prairie community [58, 82]. A comparative population genetic study of upland game birds [65] demonstrated that patch size is relevant to sustain Pheasant, Greater Prairie Chicken, and Bobwhite Quail populations, as indicated by the depressed population demographics (e.g., lower *N*_e_, higher relatedness) found in smaller habitat patches (S5 Table).

Two models are often implemented in the effort to retain/ increase grassland habitat through conversion of agricultural land. In the land-sharing model, small patches of unfarmed natural or semi-natural vegetation are retained by embedding them within larger agricultural plots, [83]. In contrast, the land-sparing model focuses on protecting/ restoring native vegetation on existing grasslands, rather than by converting agricultural land [84]. While land-sharing seems to be a strategy that is effective for species with short generation times and natural population fluctuations such as voles (i.e., they can quickly respond to changes in the landscape due to crop rotation), land-sparing is more important for longer-lived species or those with specific habitat requirements as a component of life history (e.g., leking grounds for Greater Prairie Chicken, [85]). The SAFE model of small, time-limited restoration patches (i.e., 10–15-year enrollment intervals) works well for some biodiversity components, but the retention of larger grassland patches is essential for others and should become an integral part of ecological restoration of prairie habitats to sustain larger, more demographically resilient populations [86]. Thus, restoration ’success’ will differ across species.

### Restoration beyond wildlife: A broader perspective

Benefits of ecological restoration are often aimed at providing habitat for wildlife or ensuring ecosystems functioning in an anthropogenic landscape, and are thus viewed from a biodiversity conservation stance [87, 88]. Increasingly, a broader perspective is now being applied that also considers economic impacts [89, 90] and human health [91, 92]. Financial incentives to landowners are an integral part of the SAFE initiative, but prairie habitats are also valued for their aesthetic appeal and contribution to local economies via recreation and ecotourism (e.g., bird watching, hiking, hunting) [90]. Retaining or restoring grasslands also has strong potential for natural climate mitigation [93]. Ecological restoration can benefit human health in a variety of ways, to include physiological and psychological health [92]. However, these relationships are complex and much uncertainty is involved [94].

Restoration may also have different impacts on functional connectivity of biodiversity components, including species that can contribute to spreading emerging infectious diseases (EIDs) [95, 96] In this regard, grassland rodents are recognized as important vectors and host reservoirs for EIDs, as evidenced by the contemporary spread of Hanta virus [97], as well as Lyme disease [98, 99]. In some cases, restoration reduced disease risk by diminishing the density of vectors (ticks; [100]), or pathogens (Hanta virus; [101]). In other cases, restoration practices that benefited wildlife species were also associated with higher disease risk. These can often be mitigated by targeting management of the host species (e.g., culling of deer; [102]).

In the context of grassland restoration, Lyme disease is of potential concern [103], in that the pathogen (*Borrelia burgdorferi*) is carried by a vector tick that relies upon vertebrate hosts (including voles) for dispersal [104]. Some studies have documented that an increase in tick density due to an increase in host prevalence did not associate with higher prevalence of the pathogen, and *vice-versa* [105]. In this sense, functional connectivity of the host-vector relationship may be uncoupled, and thus unpredictable. However, given the threat of Lyme disease to human health, monitoring the response of vectors to restoration efforts should be considered when benefits and risks of restoration are evaluated. An approach that coordinates agency programs, such as habitat restoration and wildlife management, can reduce potential risks [100]. Also, integrating data from different programs can inform comprehensive policies [96] and coordinate various initiatives [106].

## Conclusions

Restoring agricultural plots in midwestern North America to endemic tall grass prairie is an ongoing process implemented by federal and state agencies, but often with success difficult to quantify. Molecular approaches can provide metrics to gauge if specific restoration goals have been successfully achieved, and were applied in this study to quantify connectivity among vole populations in rehabilitated prairie patches in Illinois.

Molecular approaches can complement the ecological assessment of restoration success in multiple ways, and in addition, provide insights not obtainable otherwise. As demonstrated in this study, key aspects include genetic clarification of taxonomic uncertainty, and metrics to quantify population connectivity over larger spatial and temporal scales.

Voles in this study reflected a certain degree of demographic resilience [14, 75] as indicated by temporally stable *N*_e_ and relatedness values, despite a regional population decline in 2011. Despite this, a level of caution is warranted such that genetic data is not over-interpreted in the context of ecological inferences. Genetic connectivity, as examined in this study, does not necessarily equate to demographic connectivity [78].

Advances in molecular technologies and analyses have expanded the capacity of genetic/genomic data to address questions previously refractive. Genetic data can reveal how species respond to specific management actions (e.g., invasive species control; [107]), or test if ecological methods at the local scale (e.g., mark-recapture) can be extrapolated at the regional scale.

Collaborations between molecular and restoration ecologists can invoke a more nuanced perspective of ’restoration success.’ Genetic patterns emerge as a response to different processes, and data must be carefully evaluated [108]. In our study, genetic structure in *M*. *pennsylvanicus* was a response to social behavior (clustering of kin), and not isolation (reduced geneflow), thus mirroring observations on an iconic prairie species, the Greater Prairie Chicken [85]. Ecology must be considered along with life history when interpreting genetic patterns of study species. For example, our analyses identified potential migrants amongst SAFE sites, suggesting individual dispersal across 250km of agroecosystem—a biologically improbable event.

## Supporting information

S1 Fig Assignment of samples to species based on phenotype or genotype. Field-identification based on morphology was verified by genetic clustering, with 25% of samples reclassified.
(PDF)

S2 Fig Principal component analyses (PCA) for *M*. *ochrogaster* and *M*. *pennsylvanicus*. Genetic clusters detected in each species at Illinois SAFE sites based on PCA applied to 15 microsatellite loci.
(PDF)

S3 Fig Cross-validation of discriminant analysis of principal components (DAPC). ‘Optimal’ number of retained principal component (PC) axes (X-axis) based on minimum of root-mean-square-error (RMSE) assignment (Y-axis) of 20% of randomly selected samples.
(PDF)

S4 Fig Optimal number of genetic clusters in *M*. *ochrogaster* and *M*. *pennsylvanicus*. Evaluation of 10 replicates at *K* values 1–10 based on the ’ad hoc statistic’ Δ*K* and the probability of *K*.
(PDF)

S5 Fig Power analysis *M*. *ochrogaster* and *M*. *pennsylvanicus*. Ability to resolve population structure as evaluated with two statistical measures (χ2 and Fisher exact tests).
(PDF)

S6 Fig Temporal stability of effective population size *N*_e_ in *M*. *ochrogaster*. Comparison of *N*_e_ estimates and 95% jackknife confidence intervals between years and two SAFE sites.
(PDF)

S1 Table SAFE study sites and restoration patches. Details on geographic location, patch area, acronyms and sample sizes based on phenotypic and genetic identifications.
(PDF)

S2 Table Microsatellite DNA loci. Details on primers used to amplify 23 microsatellite loci.
(PDF)

S3 Table Pairwise *F*_ST_ estimates for *M*. *ochrogaster*. Values calculated for 2010 and 2012 between restoration patches.
(PDF)

S4 Table Average relatedness *r* for *M*. *ochrogaster*. Estimates of within-patch average and variance by year.
(PDF)

S5 Table Genetic diversity and connectivity across five prairie species. Comparison of population genetic and life history parameters for two vole and three bird species.
(PDF)
