# Supplementary material for: Population connectivity in voles (Microtus sp.) as a gauge for tall grass prairie restoration in midwestern North America
Source: PLoS One. 2021 Dec 9;16(12):e0260344. doi: 10.1371/journal.pone.0260344 (PMC8659414; doi:10.1371/journal.pone.0260344)
Supplement: S1 Fig — Field-identification based on morphology was verified by genetic clustering, with 25% of samples reclassified. (PDF) [file pone.0260344.s001.pdf]

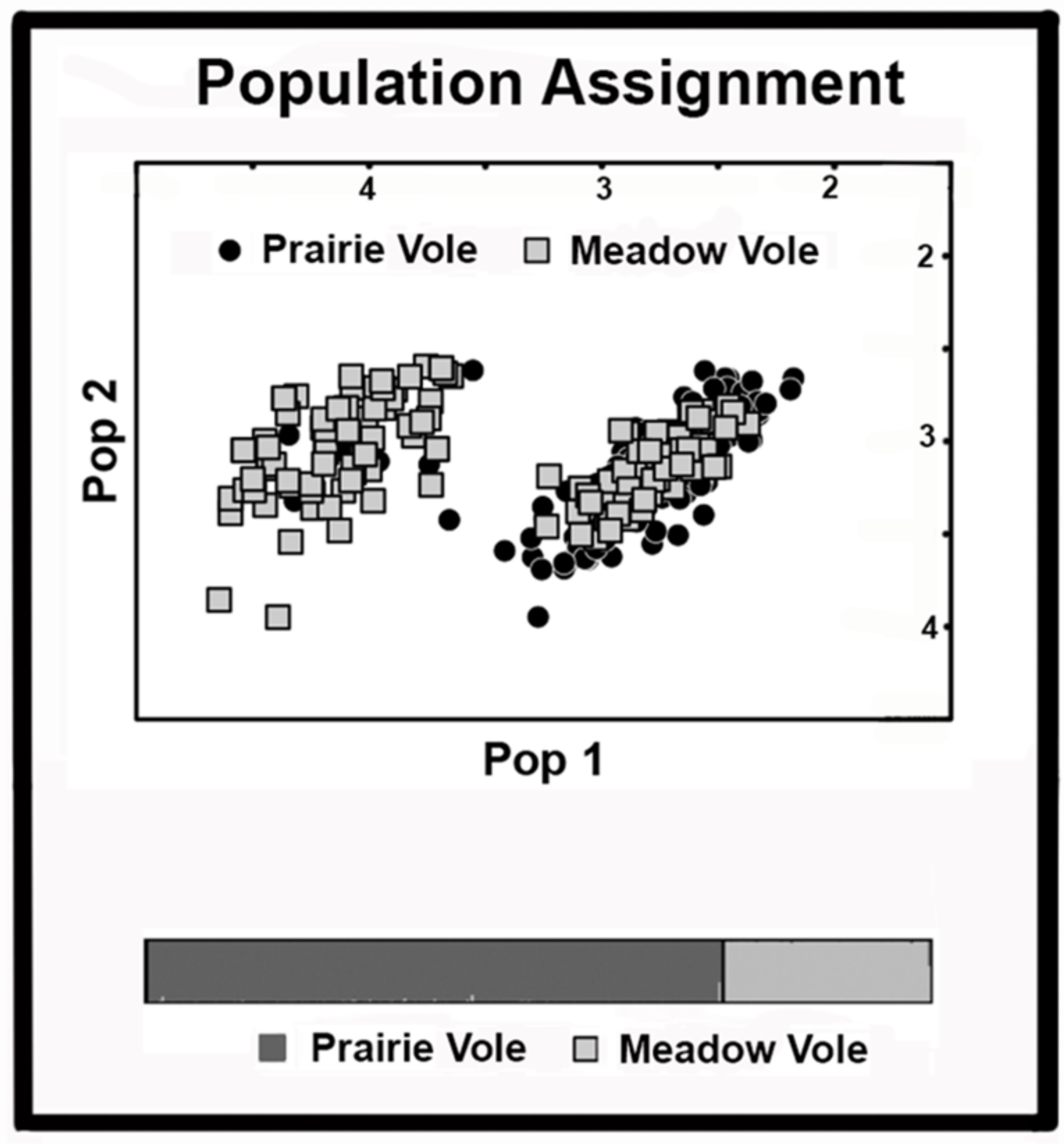

S1 Figure. Allocation of *M. ochrogaster* (prairie vole) and *M. pennsylvanicus* (meadow vole) samples to species. Samples (*N*=314) were collected at five SAFE sites in Illinois from 2010–2012. Top: Initial field-based species identification utilized morphology. Multilocus genotypes across 15 microsatellite loci were then used to allocate individuals to two gene pools using a population assignment test (GenAlEx v. 6.5). Bottom: Species identification based on molecular genetic reclassification. Allocation of individuals to two gene pools using Bayesian clustering (Structure 2.3.4.). Shading reflects distinct gene pools and vertical bars represent probabilities of assigning an individual to a gene pool.

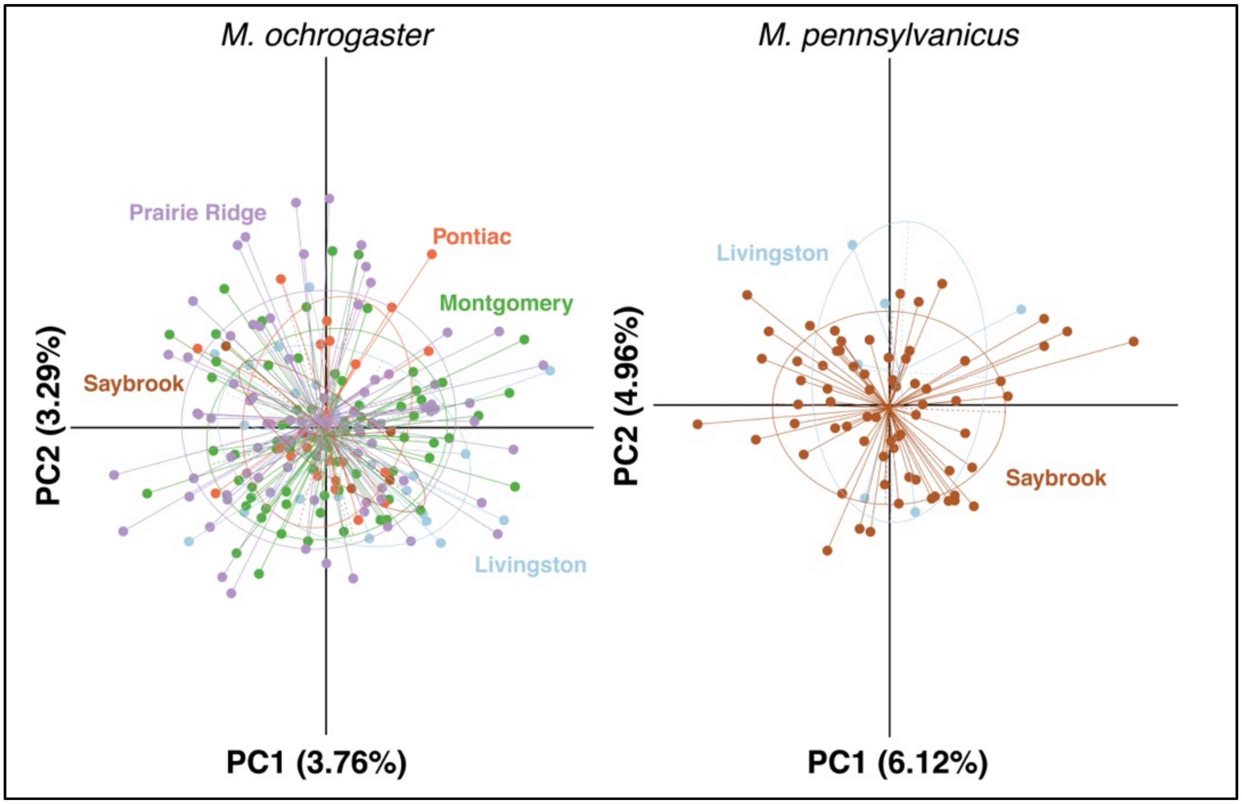

S2 Figure. Principal component analyses (PCA) for *M. ochrogaster* and *M. pennsylvanicus* representing genetic clusters found in five Illinois SAFE sites (=Livingston, Montgomery, Pontiac, Prairie Ridge, and Saybrook) in the former and two sites in the latter (=Livingston and Saybrook). Plots are based on PCA applied to 15 microsatellite loci. Colors reflect unique SAFE sites and are consistent across species; individuals are represented as points. The percentage of variation from the discriminant analysis captured by each PC axis is provided in parentheses. Plots depict only the first two PC axes in each species.

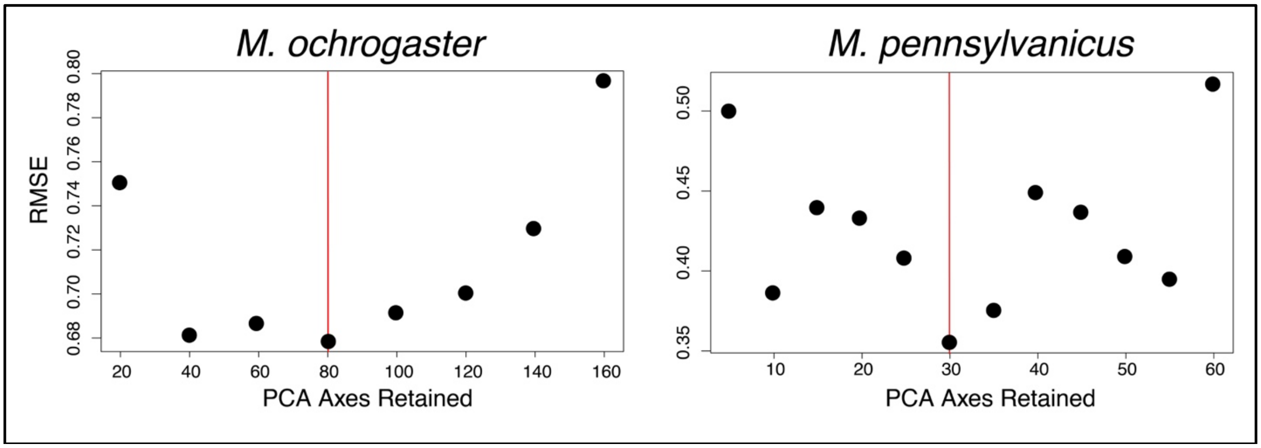

S3 Figure. Discriminant analysis of principal components (DAPC) cross-validation results for *M. ochrogaster* and *M. pennsylvanicus* with the Y-axis showing the root-mean-square-error (RMSE) of assignment for a 20% test set of randomly selected samples across a range of retained principal component (PC) axes (X-axis); the 'optimal' number of PCs was selected as that which minimized RMSE.

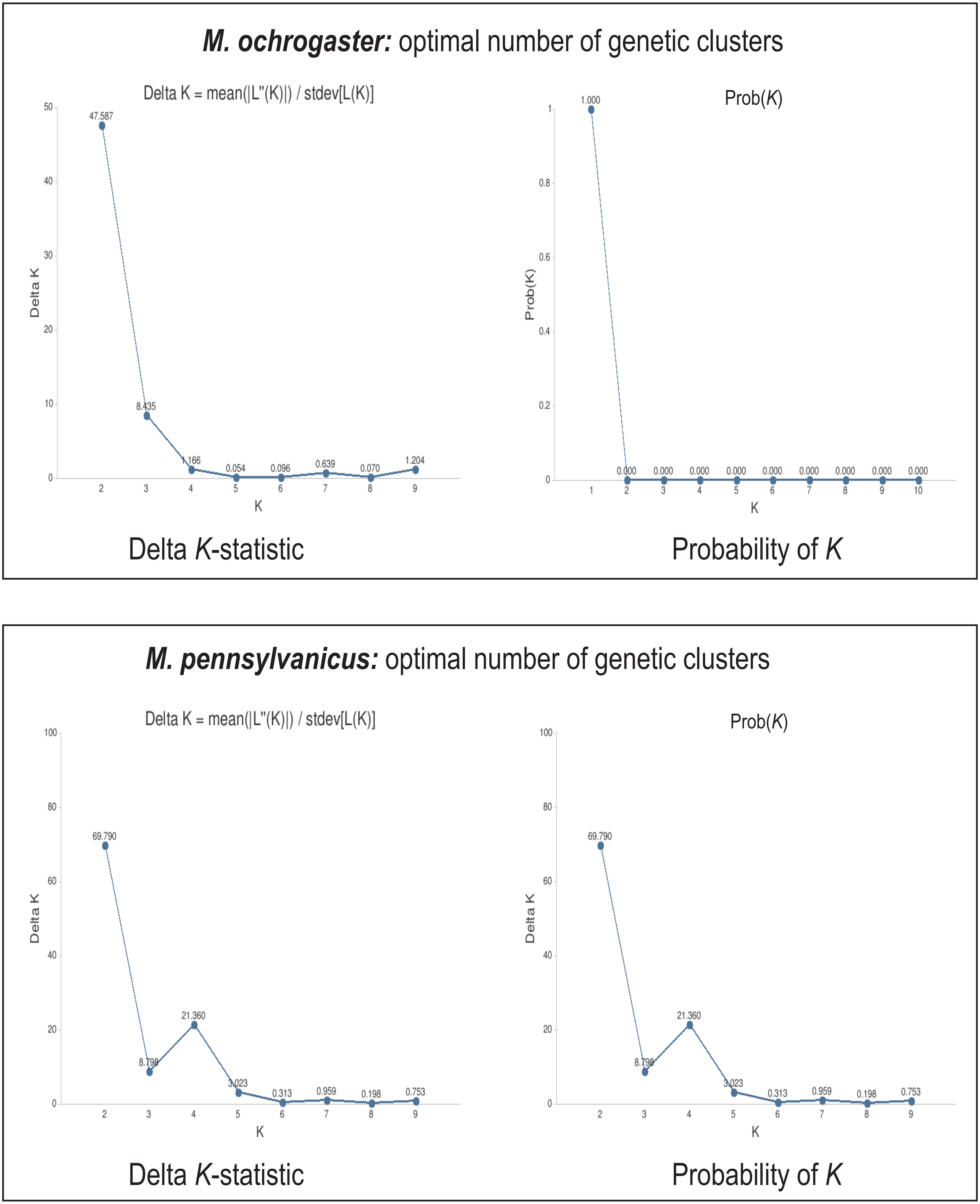

S4 Fig. Optimal number of genetic clusters in *M. ochrogaster* and *M. pennsylvanicus*.
Evaluation of 10 replicates at *K* values 1–10 based on the 'ad hoc statistic' Δ*K* and the probability of *K*.

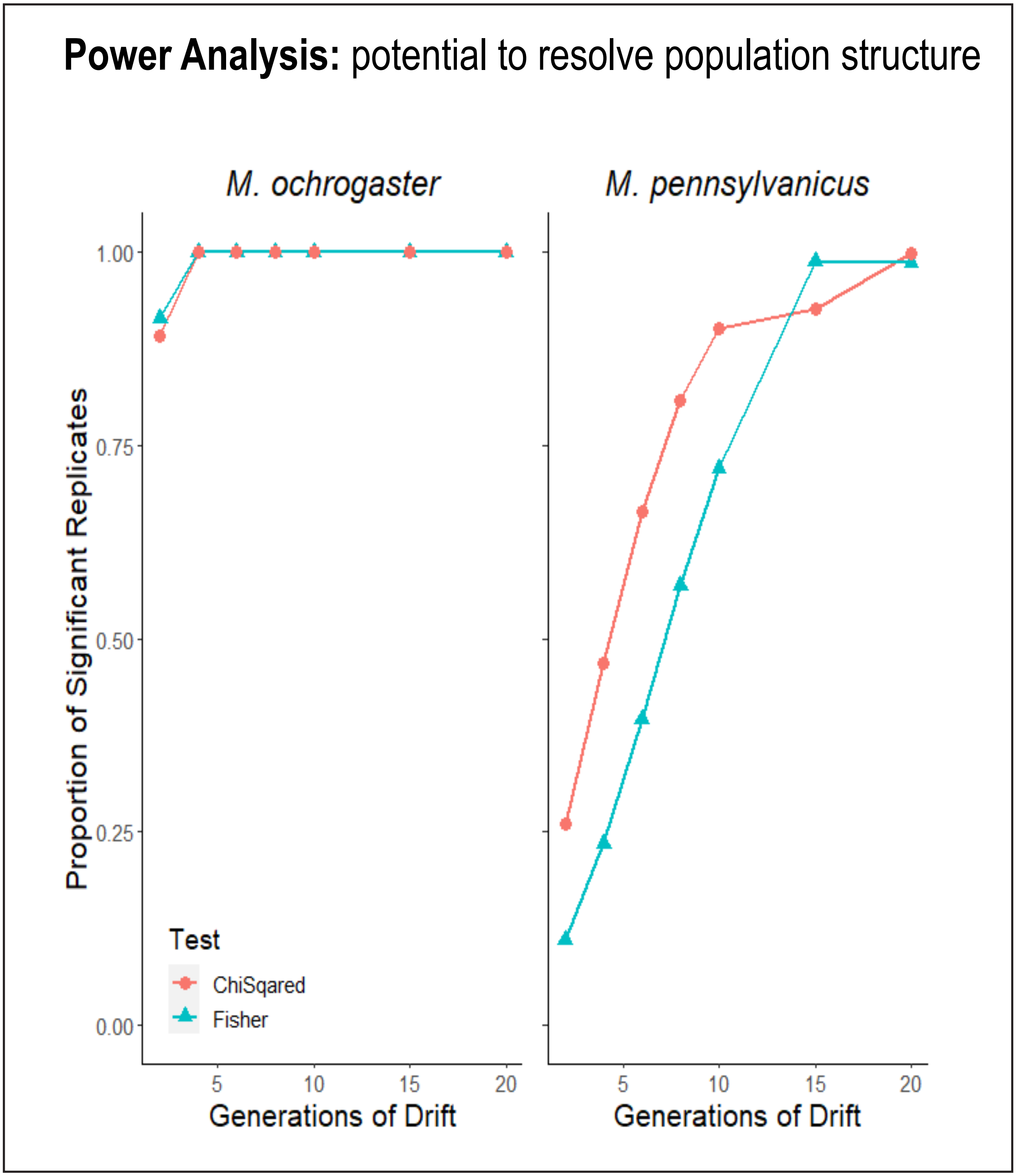

S5 Fig. Power analysis *M. ochrogaster* and *M. pennsylvanicus.*
Ability to resolve population structure as evaluated with two statistical measures (χ2 and Fisher exact tests).

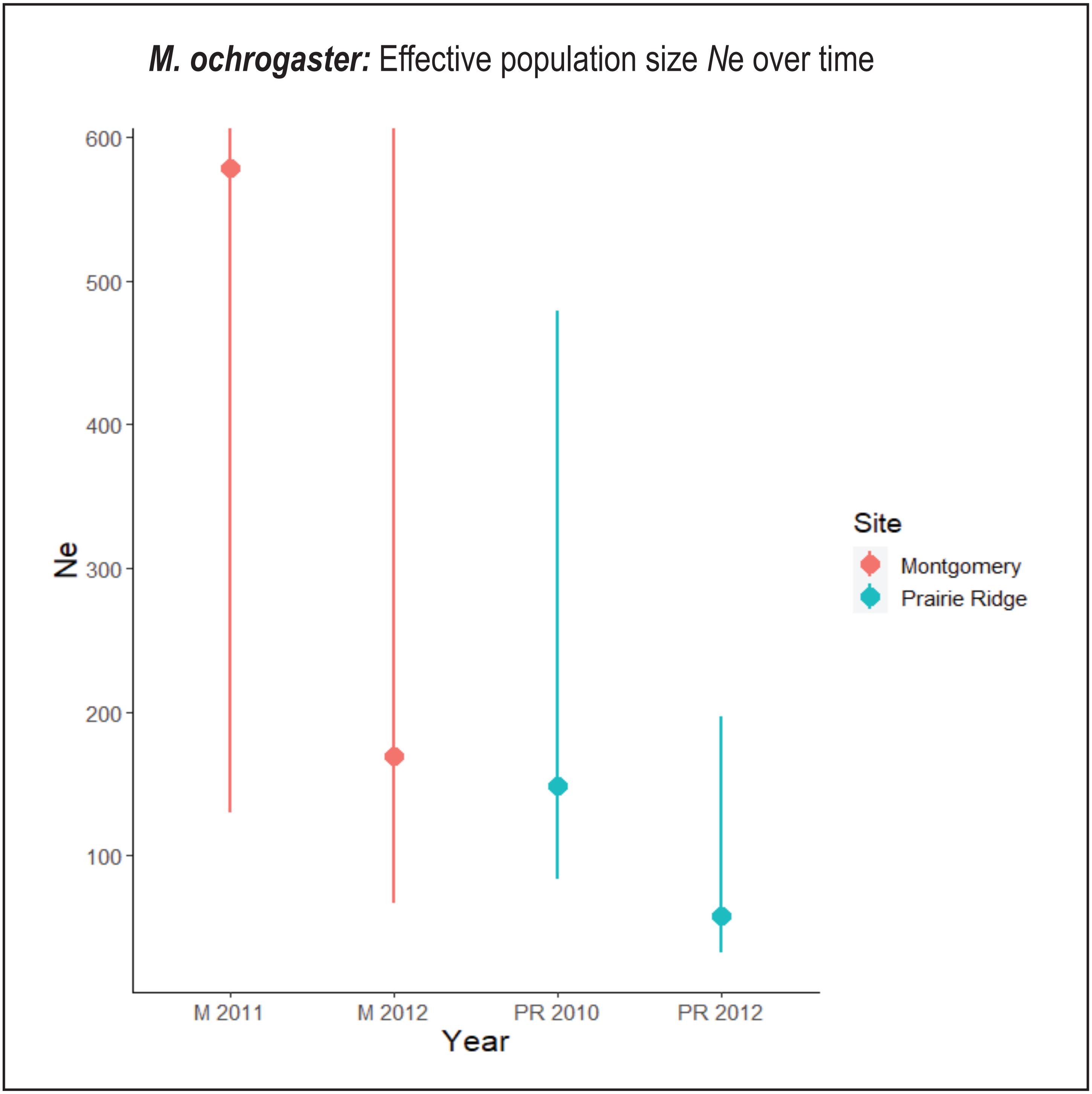

S6 Fig. Temporal stability of effective population size *Ne* in *M. ochrogaster*
Comparison of *Ne* estimates and 95% jackknife confidence intervals between years and two SAFE sites.
