# Supplementary material for: Population connectivity in voles (Microtus sp.) as a gauge for tall grass prairie restoration in midwestern North America
Source: PLoS One. 2021 Dec 9;16(12):e0260344. doi: 10.1371/journal.pone.0260344 (PMC8659414; doi:10.1371/journal.pone.0260344)
Supplement: S2 Fig — Genetic clusters detected in each species at Illinois SAFE sites based on PCA applied to 15 microsatellite loci. (PDF) [file pone.0260344.s002.pdf]

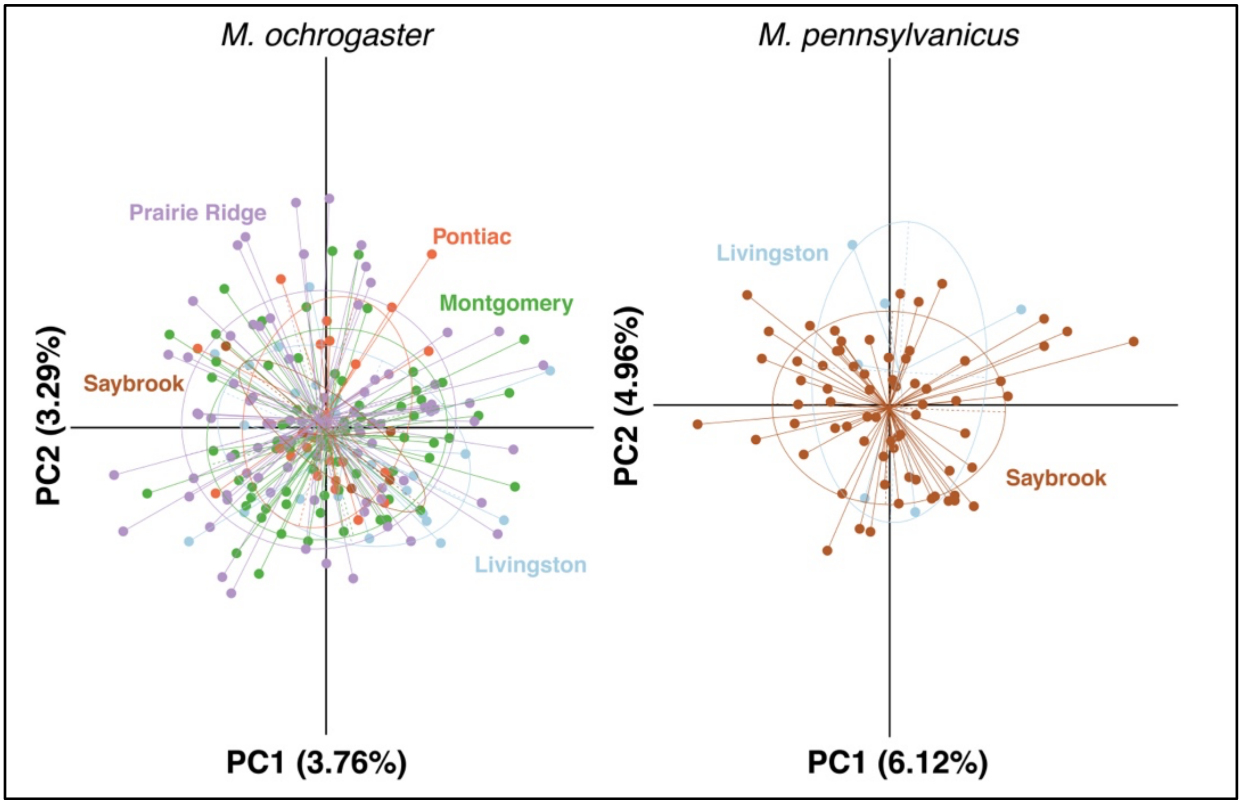

S2 Figure. Principal component analyses (PCA) for *M. ochrogaster* and *M. pennsylvanicus* representing genetic clusters found in five Illinois SAFE sites (=Livingston, Montgomery, Pontiac, Prairie Ridge, and Saybrook) in the former and two sites in the latter (=Livingston and Saybrook). Plots are based on PCA applied to 15 microsatellite loci. Colors reflect unique SAFE sites and are consistent across species; individuals are represented as points. The percentage of variation from the discriminant analysis captured by each PC axis is provided in parentheses. Plots depict only the first two PC axes in each species.
