# Supplementary material for: Population connectivity in voles (Microtus sp.) as a gauge for tall grass prairie restoration in midwestern North America
Source: PLoS One. 2021 Dec 9;16(12):e0260344. doi: 10.1371/journal.pone.0260344 (PMC8659414; doi:10.1371/journal.pone.0260344)
Supplement: S3 Fig — ‘Optimal’ number of retained principal component (PC) axes (X-axis) based on minimum of root-mean-square-error (RMSE) assignment (Y-axis) of 20% of randomly selected samples. (PDF) [file pone.0260344.s003.pdf]

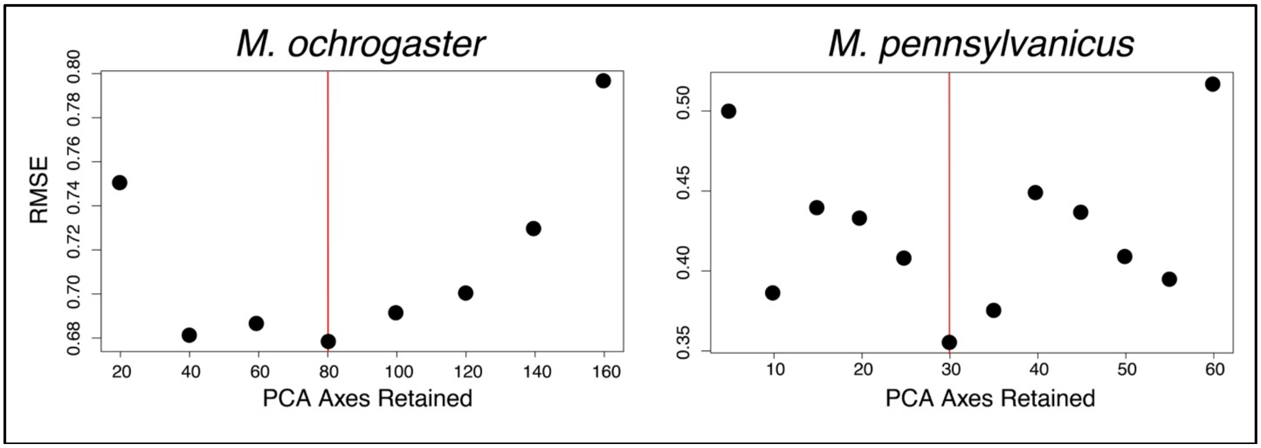

S3 Figure. Discriminant analysis of principal components (DAPC) cross-validation results for *M. ochrogaster* and *M. pennsylvanicus* with the Y-axis showing the root-mean-square-error (RMSE) of assignment for a 20% test set of randomly selected samples across a range of retained principal component (PC) axes (X-axis); the 'optimal' number of PCs was selected as that which minimized RMSE.
