# Supplementary material for: Population connectivity in voles (Microtus sp.) as a gauge for tall grass prairie restoration in midwestern North America
Source: PLoS One. 2021 Dec 9;16(12):e0260344. doi: 10.1371/journal.pone.0260344 (PMC8659414; doi:10.1371/journal.pone.0260344)
Supplement: S4 Fig — Evaluation of 10 replicates at K values 1–10 based on the ’ad hoc statistic’ ΔK and the probability of K. (PDF) [file pone.0260344.s004.pdf]

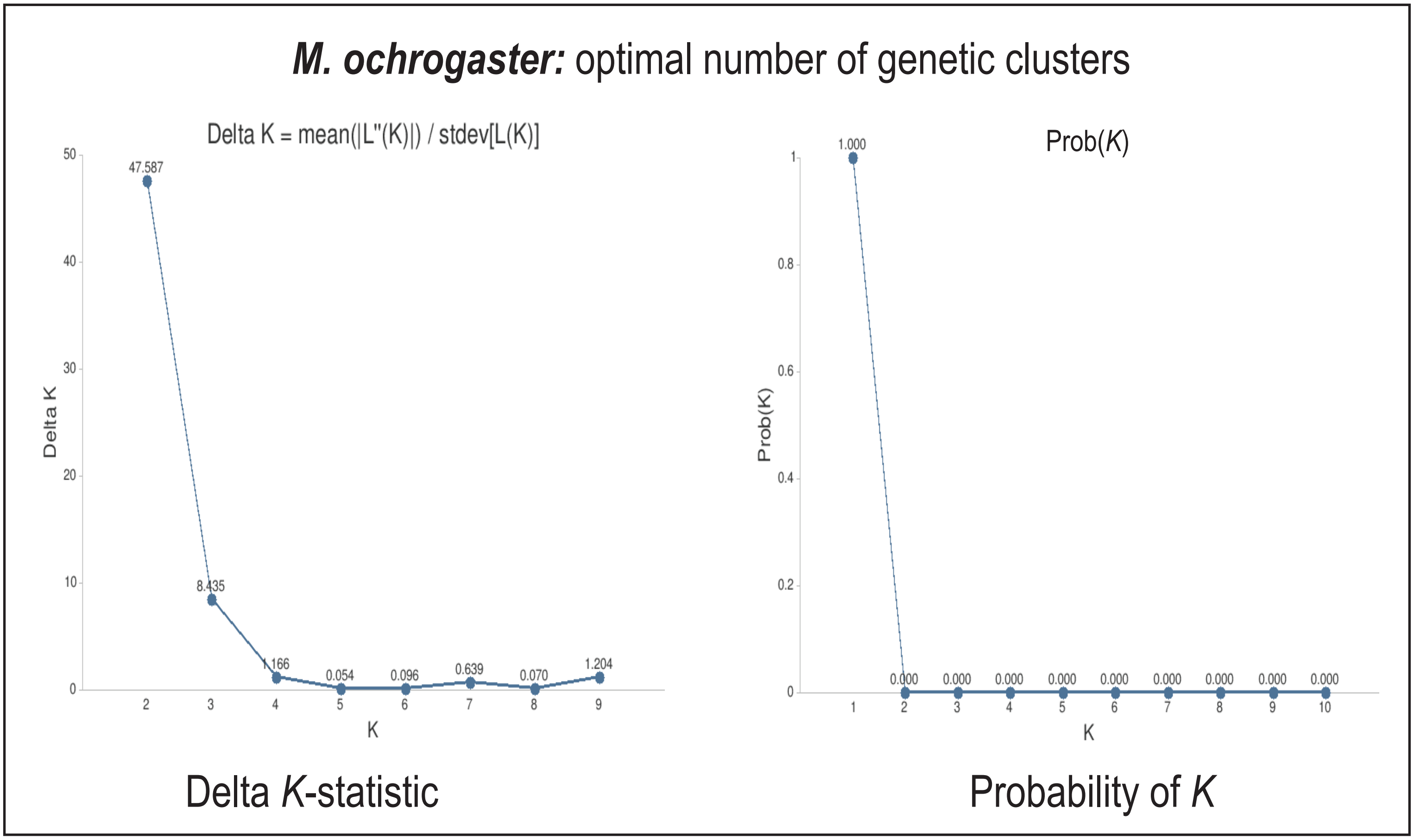

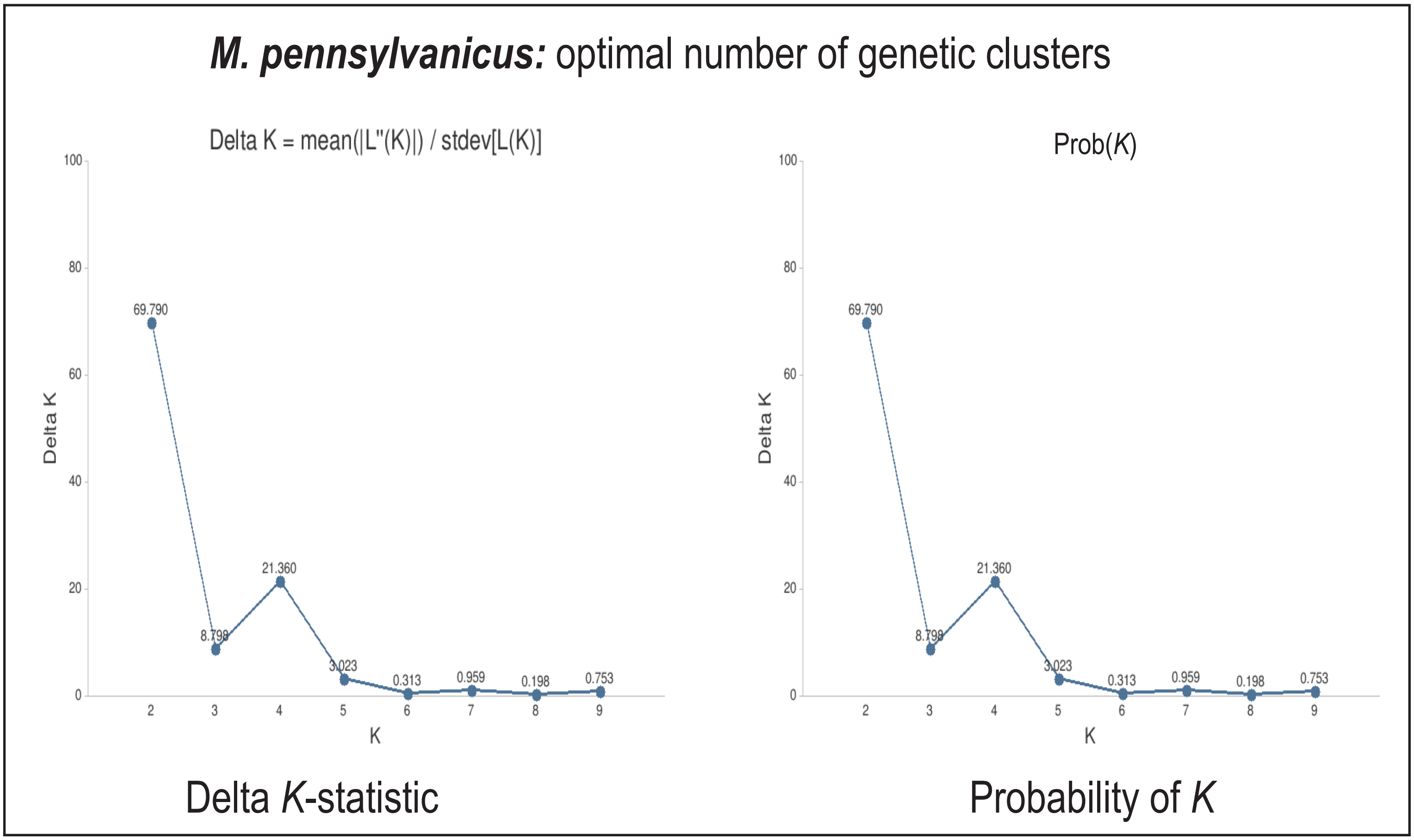

S4 Fig. Optimal number of genetic clusters in *M. ochrogaster* and *M. pennsylvanicus*.
Evaluation of 10 replicates at *K* values 1–10 based on the 'ad hoc statistic' Δ*K* and the probability of *K*.
