# Supplementary material for: Population connectivity in voles (Microtus sp.) as a gauge for tall grass prairie restoration in midwestern North America
Source: PLoS One. 2021 Dec 9;16(12):e0260344. doi: 10.1371/journal.pone.0260344 (PMC8659414; doi:10.1371/journal.pone.0260344)
Supplement: S5 Fig — Ability to resolve population structure as evaluated with two statistical measures (χ2 and Fisher exact tests). (PDF) [file pone.0260344.s005.pdf]

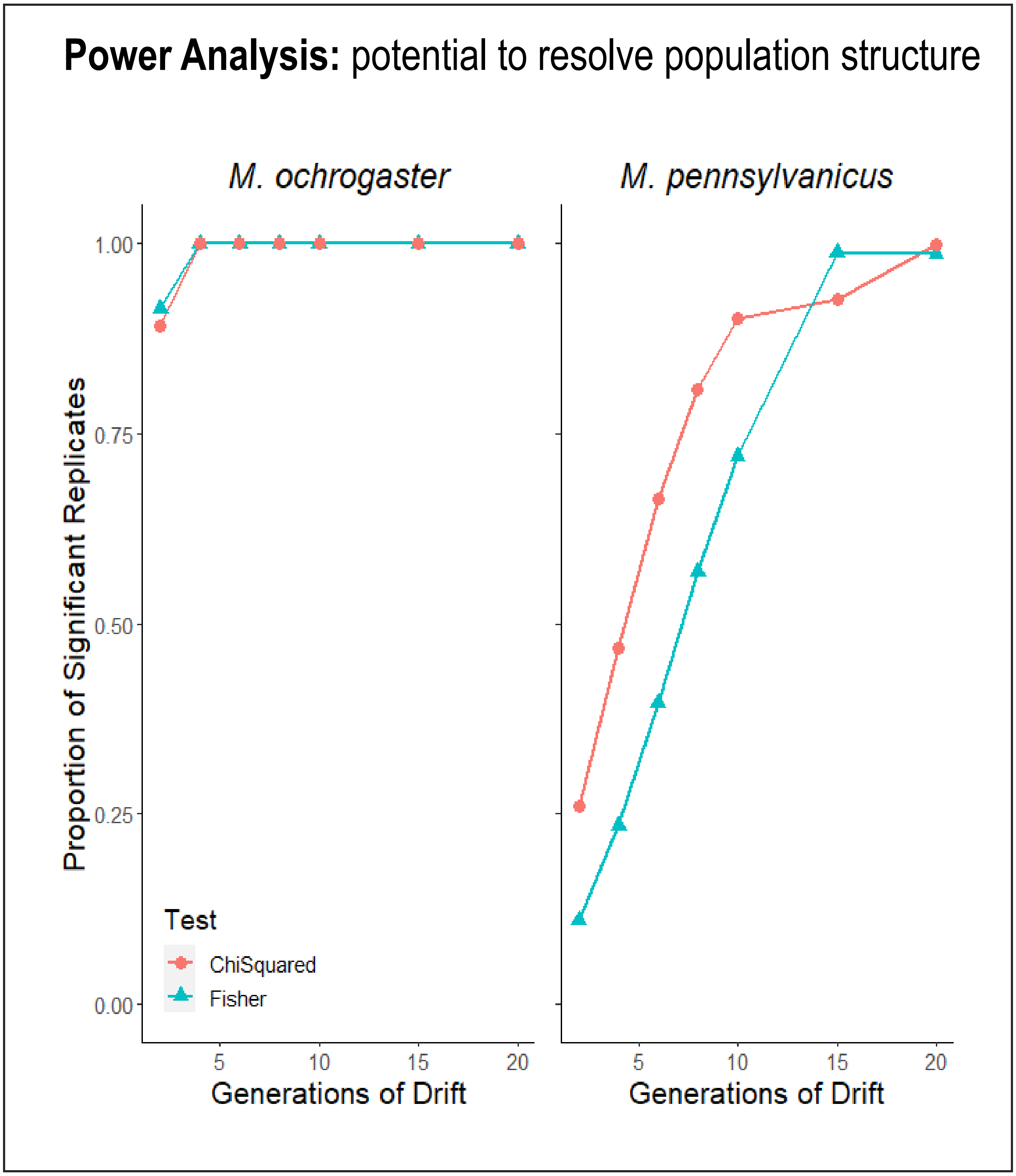

S5 Fig. Power analysis *M. ochrogaster* and *M. pennsylvanicus.*
Ability to resolve population structure as evaluated with two statistical measures (χ2 and Fisher exact tests).
