# Supplementary material for: Population connectivity in voles (Microtus sp.) as a gauge for tall grass prairie restoration in midwestern North America
Source: PLoS One. 2021 Dec 9;16(12):e0260344. doi: 10.1371/journal.pone.0260344 (PMC8659414; doi:10.1371/journal.pone.0260344)
Supplement: S6 Fig — Comparison of Ne estimates and 95% jackknife confidence intervals between years and two SAFE sites. (PDF) [file pone.0260344.s006.pdf]

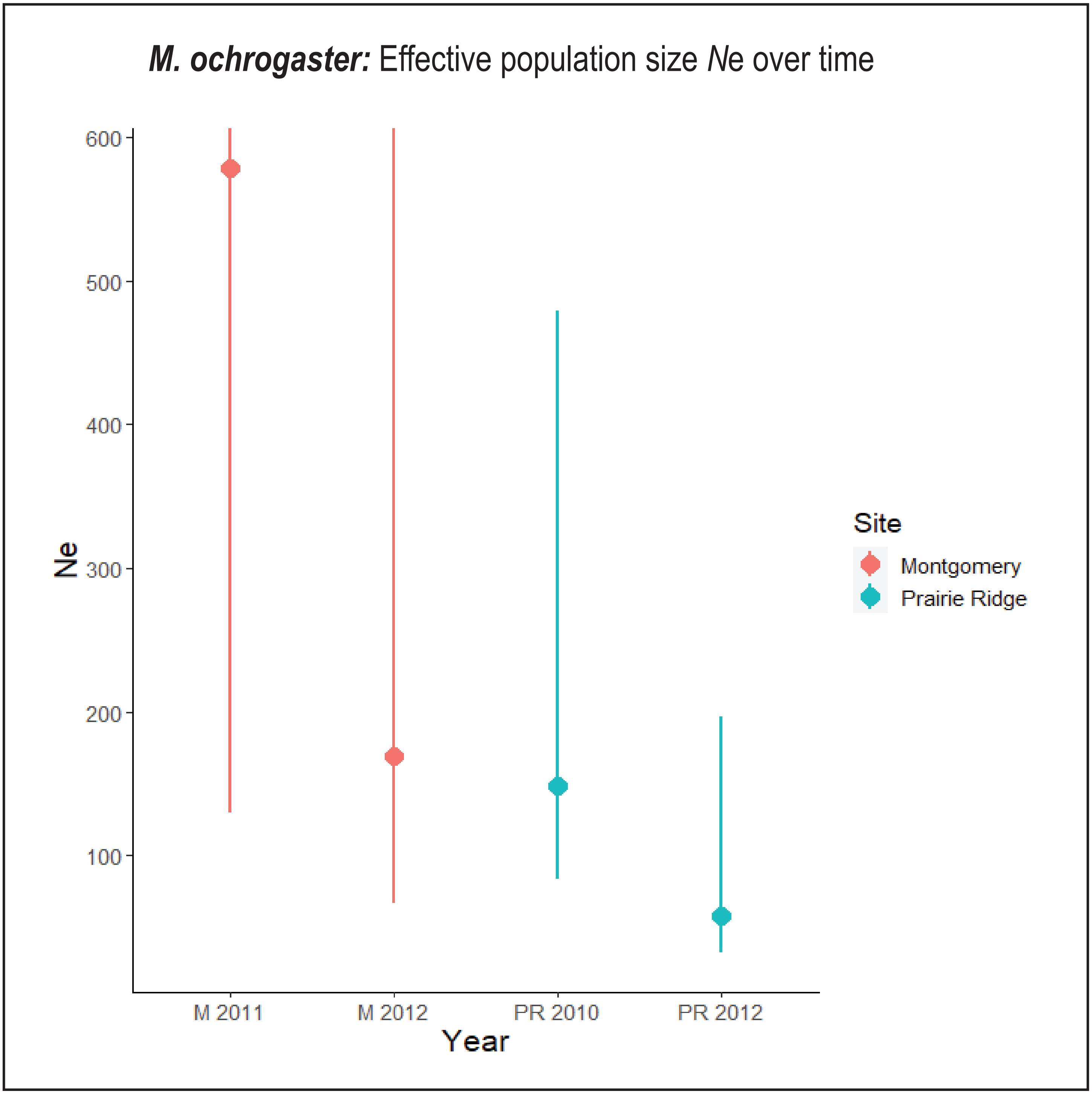

S6 Fig. Temporal stability of effective population size *Ne* in *M. ochrogaster*
Comparison of *Ne* estimates and 95% jackknife confidence intervals between years and two SAFE sites.
