# Supplementary material for: Population connectivity in voles (Microtus sp.) as a gauge for tall grass prairie restoration in midwestern North America
Source: PLoS One. 2021 Dec 9;16(12):e0260344. doi: 10.1371/journal.pone.0260344 (PMC8659414; doi:10.1371/journal.pone.0260344)
Supplement: S1 Table — Details on geographic location, patch area, acronyms and sample sizes based on phenotypic and genetic identifications. (PDF) [file pone.0260344.s007.pdf]

S1 Table: SAFE study sites and patches

SAFE sites and restoration patches within each listed by County. Provided for each patch are the following: Area = size of restoration patch (in ha); Latitude, Longitude = coordinates for geographic location; Code = acronym used to designate samples from each patch. Numbers of samples representing each species are listed as identified based on phenotype (FieldID) or genotype (GenID), with the MIOC = *M. ochrogaster* (prairie vole) and MIOC *M. pennsylvanicus* (the meadow vole). Locations of SAFE sites within Illinois are depicted in Figure 1.

County	SAFE Site	Patch	Area (ha)	Latitude	Longitude	Code	FieldID		GenID	
							MIOC	MIPE	MIOC	MIPE
Livingston	**Pontiac**	Curve	37.19	41º 4' N	88º 42' E	POCU	10	-	10	-
Livingston	**Pontiac**	Treeline	20.31	41º 1' N	88º 38' E	POTR	9	-	9	-
Livingston	**Pontiac**	Tower	1.96	41º 1' N	88º 40' E	POTO	6	-	6	-
Livingston	**Livingston**	Hummel	51.1	40º 42' N	88º 15' E	LIHU	14	2	16	-
Livingston	**Livingston**	Marge	61.54	40º 41' N	88º 16' E	LIMA	13	3	13	3
Livingston	**Livingston**	Dasso	6.48	40º 40' N	88º 17' E	LIDA	-	3	-	3
McLean	**Saybrook**	Radio North	58.86	40º 29' N	88º 33' E	SARN	2	4	2	5
McLean	**Saybrook**	Radio South	64.85	40º 29' N	88º 33' E	SARS	1	1	-	1
McLean	**Saybrook**	Anchor North	27.28	40º 29' N	88º 31' E	SAAN	3	11	3	11
McLean	**Saybrook**	Anchor South	8.46	40º 29' N	88º 31' E	SAAS	2	9	-	11
McLean	**Saybrook**	Anchor West	20.31	40º 29' N	88º 31' E	SAAW	5	22	-	27
McLean	**Saybrook**	Old Say	27.05	40º 27' N	88º 33' E	SAOS	-	22	-	22
Montgomery	**Montgomery**	Lane	15.74	39º 8' N	89º 26' E	MOLA	14	18	32	-
Montgomery	**Montgomery**	Huber South	27.31	39º 7' N	89º 23' E	MOHU	40	9	49	-
Jasper	**Prairie Ridge**	Tombstone	7.29	38º 56' N	88º 11' E	PRTO	26	26	52	-
Jasper	**Prairie Ridge**	Harvey	15.74	38º 53' N	88º 9' E	PRHA	27	10	37	-
Jasper	**Prairie Ridge**	BW	38.67	38º 53' N	88º 11' E	PRBW	2	-	2	-
						**Total by species**	**174**	**140**	**231**	**83**
