# Supplementary material for: Population connectivity in voles (Microtus sp.) as a gauge for tall grass prairie restoration in midwestern North America
Source: PLoS One. 2021 Dec 9;16(12):e0260344. doi: 10.1371/journal.pone.0260344 (PMC8659414; doi:10.1371/journal.pone.0260344)
Supplement: S2 Table — Details on primers used to amplify 23 microsatellite loci. (PDF) [file pone.0260344.s008.pdf]

S2 Table: Microsatellite DNA Loci

Details of 23 microsatellite DNA loci successfully amplified in *M. ochrogaster* and *M. pennsylvanicus*. Loci were combined into six multiplex panels for data generation. Listed for each locus: Primer Name = published primer name; ABI Dye = fluorescent dye used to label forward primer; Motif = repeat motif; Citation = source of primer; Forward/ Reverse Primer = primer sequences. Amplifications were conducted in 10–15µl volume polymerase chain reactions (PCR) using 1X *Flexi*-buffer (PROMEGA), 2.5–3.5mM $MgCl_2$, 0.25mM dNTPs, 0.2µg BSA, 1 unit *Go*-taq polymerase (PROMEGA), 0.1µM of each forward and reverse primer, and approximately 10–15ng template DNA. Reactions were carried out under the following conditions: initial denaturation at 94°C for 3min, followed by 15 cycles of 94°C for 45s, 55°C annealing temperature for 45s, and a 72°C extension for 30s, followed by an additional 25 cycles of 95°C for 30s, 55°C annealing temperature for 30s, and a 72°C extension for 15s, followed by a final extension at 72°C for 3min.

Multiplex	Primer Name	ABI Dye	Motif	Citation	Forward Primer	Reverse Primer
1	AV14	6-FAM	$(GATA)_{16}$	Stewart et al. 1998	TATGTGATATGGCACTAGCATGT	AGCCTGTCTCAGCAGAAGG
	Ma35	VIC	$(GT)_{18}$	Gauffre et al. 2007	AGAGTATGGCTGAGGGTG	GCCAGAGCAGTGTGATG
	AV15	NED	$(GATA)_{14}$	Stewart et al. 1998	TATATGGAAGGTCGTAGATTCAG	ATTAAAGCATTTGTTGAGAAAGC
	AV13	PET	$(GATA)_{14}$	Stewart et al. 1998	CTGGCTCTATCTATCTGTCTATC	ACAATTACAGCATCCAGAAG
2	AT23	6-FAM	$(TG)_{20}$	Berthier et al. 2004	GGATCATCTTCGCTAAGGAG	CCATCTCAGGCCTAATTCAG
	MSMM-3	VIC	(CA)15	Ishibashi et al. 1999	TACGCCCCTTCAAACTCATGTG	TCCTTTATCTTAGGTGATGGAG
	MSMM-2	NED	$(CA)_{21}(GA)_{22}$	Ishibashi et al. 1999	TAACCACAACCCCTCCAACTG	TCATTTGGAGTTGCTGAGAAC
	MSMM-6	PET	$(CA)_{18}$	Ishibashi et al. 1999	TACAAATCTATCCTCTGACCTC	TACAAAGCCATTGTTCCCTGCT
3	Mar076	VIC	$(AC)_{16}$	Walser and Heckel 2008	TCACCAGGACCTACTGAGCA	GCCAGCTTCATTTCAAGAGG
	Cg17A7	NED	$(ATGT)_{9}$	Rikalainen et al. 2008	ACATTCAAACCTATGGGACA	GAAGGCTATTGATCTTGCAC
	Ma66	PET	$(TG)_{24}$	Gauffre et al. 2007	AAGGTCTGGTGGATGTCAGG	TGCAAGGCAGGATTCTACC
4	LIST3-005	PET	$(GT)_{21}$	Barker et al. 2005	ATGAGGCTTCTTTCTATGTCC	CCTGCTCTGTATGCTTTGA
	Mar113	6-FAM	$(AC)_{12}$	Walser and Heckel 2008	AAGAGCCTGCTGTGGTTTGT	TCAGCTGGGAATCAGGTCTT
	Mar003	VIC	$(TG)_{21}$	Walser and Heckel 2008	GGAGATACAAGGCCCAAACA	TGGCATTAGATGACCTGTGG
	Ma-09	NED	$(AC)_{18}$	Gauffre et al. 2007	CCCTAAGGAATAGCATCTGAG	GAATGTATGTGGAAGCCAGG
	Mar016	PET	$(CA)_{19}$	Walser and Heckel 2008	CATCATCTTCTGGGGCACTG	ACGGTCTGTGCAAACCACTT
5	MAG25	6-FAM	$(CA)_{17}$	Jaarola et al. 2007	TGGGATAGCCTAGCAGCAAGA	GTTTGTAGGGTTAGGTTCTCAGTTG
	AT2	VIC	$(TC)_{20}$	Berthier et al. 2004	CAAAGAGGAAGTGCTAGGTTGG	ACCCTTGGGACTCTGTTTGC
	Mar063	PET	$(AC)_{23}$	Walser and Heckel 2008	GCCTGGACACAACCAAACTT	GGCTATGGGCAGCTCCTG
6	MSMM-5	6-FAM	$(CA)_{17}$	Ishibashi et al. 1999	TCTAATACCCTCTTCCTTGGG	TCCTATCAAGGGGCATTCATCT
	MAG18	VIC	$(AC)_{22}$	Jaarola et al. 2007	GTATGCCTGCTATTGTGAAGAC	GTTTGCTCTTTTGCTCCCAGTAACTCAC
	MAG26	NED	$(AG)_{31}$	Jaarola et al. 2007	CCTCTCAAAGCAGTTAAGC	GTTTAGCGTTACTATTGTAGCC
	MSMM-4	PET	$(CA)_{19}$	Ishibashi et al. 1999	TGTTTCAAGGCAATAAGGTGG	TCGTTTCCCTGGAGATTGGG

References Cited:

Barker FS, Helyar J, SJ Kemp, Begon M. Highly polymorphic microsatellite loci in the bank vole (*Clethrionomys glareolus*). Mol Ecol Notes. 2005; 5: 311–313. https://doi.org/10.1111/j.1471-8286.2005.00911.x

Berthier K, Galan M. Weber A, Loiseau A, Cosson JF. A multiplex panel of dinucleotide microsatellite markers for the water vole, *Arvicola terrestris.* Mol Ecol Notes. 2004; 7: 620–622. https://doi.org/10.1111/j.1471-8286.2004.00756.x

Gauffre B, Galan M, Bretagnolle V, Cosson JF. Polymorphic microsatellite loci and PCR multiplexing in the common vole, *Microtus arvalis*. Mol. Ecol. Notes 2007; 7: 830–832. https://doi.org/10.1111/j.1471-8286.2007.01718.x

Ishibashi Y, Yoshinaga Y, Saitoh T, Abe S, Iida H, Yoshida MC. Polymorphic microsatellite DNA markers in the field vole *Microtus montebelli.* Mol Ecol. 1999; 8: 163–164. PMID: 9919707

Jaarola M, Ratkiewicz M, Ashford RT, Brunhoff C, Borkowksa A. Isolation and characterization of polymorphic microsatellite loci in the field vole, *Microtus agrestis*, and their cross-utility in the common vole, *Microtus arvalis.* Mol Ecol Notes. 2007; 7: 1029–1031. https://doi.org/10.1111/j.1471-8286.2007.01763.x

Rikalainen K, Grapputo A, Knott E, Koskela E, Mappes T. A large panel of novel microsatellite markers for the bank vole (*Myodes glareolus*). Mol Ecol Res. 2008; 8: 1164–1168. https://doi.org/10.1111/j.1755-0998.2008.02228.x

Stewart WA, Piertney WA, Dallas DF. Isolation and characterization of highly polymorphic microsatellites in the water vole, *Arvicola terrestris.* Mol Ecol. 1998; 7: 1247–1263. PMID: 9734085

Walser B, Heckel G. Microsatellite markers for the common vole (*Microtus arvalis*) and their cross-species utility. Cons Gen. 2008; 9: 479–481. https://doi.org/10.1007/s10592-007-9355-6
