# Supplementary material for: Population connectivity in voles (Microtus sp.) as a gauge for tall grass prairie restoration in midwestern North America
Source: PLoS One. 2021 Dec 9;16(12):e0260344. doi: 10.1371/journal.pone.0260344 (PMC8659414; doi:10.1371/journal.pone.0260344)
Supplement: S3 Table — Values calculated for 2010 and 2012 between restoration patches. (PDF) [file pone.0260344.s009.pdf]

S3 Table: Pairwise F_{ST}

S3 Table. Pairwise F_{ST} values for *M. ochrogaster* by year between restoration patches collect at SAFE sites (= Site) in 2010 (upper panel) and 2012 (lower panel). Data were derived from 15 microsatellite DNA loci. F_{ST} values are below the diagonal and *p*-values above the diagonal. Bonferroni-adjusted statistical significance: 2010 $p < 0.003$ and 2012 $p < 0.008$. Significant values are in bold.

2010 - Site	Patch	L-H	L-M	P-C	P-T	PR-H	PR-T
Livingston	Hummel	X	0.893	0.024	0.475	0.079	**0.002**
Livingston	Marge	0.020	X	0.601	0.783	0.580	**0.001**
Pontiac	Curve	0.031	0.028	X	0.923	0.156	**0.001**
Pontiac	Tower	0.037	0.036	0.036	X	0.534	0.090
Prairie Ridge	Harvey	0.017	0.017	0.022	0.029	X	**0.001**
Prairie Ridge	Tombstone	0.025	0.029	0.033	0.038	0.018	X

2012 - Site	Patch	M-H	M-L	PR-H	PR-T
Montgomery	Huber	X	0.023	**0.006**	0.012
Montgomery	Lane	0.025	X	0.011	**0.001**
Prairie Ridge	Harvey	0.055	0.044	X	0.013
Prairie Ridge	Tombstone	0.027	0.023	0.043	X
