# Supplementary material for: Population connectivity in voles (Microtus sp.) as a gauge for tall grass prairie restoration in midwestern North America
Source: PLoS One. 2021 Dec 9;16(12):e0260344. doi: 10.1371/journal.pone.0260344 (PMC8659414; doi:10.1371/journal.pone.0260344)
Supplement: S4 Table — Estimates of within-patch average and variance by year. (PDF) [file pone.0260344.s010.pdf]

S4 Table: Average relatedness (*r*)

S4 Table. Average relatedness (*r*) in *M. ochrogaster* by year and patch. Data were derived from 15 microsatellite DNA loci. Samples are listed by year for each SAFE site and restoration patch for which estimates could be obtained. *N* = numbers of samples by patch; *r* = average relatedness; Variance = variance of average relatedness.

Site	Patch	*N*	*r*	Variance
**2010**				
Livingston	Hummel	13	0.037	0.004
Livingston	Marge	11	0.027	0.002
Pontiac	Curve	9	0.025	0.001
Pontiac	Tower	5	0.009	0.000
Prairie Ridge	Harvey	28	0.031	0.004
Prairie Ridge	Tombstone	26	0.050	0.007
**2011**				
Montgomery	Huber	34	0.027	0.006
Montgomery	Lane	8	0.058	0.015
**2012**				
Montgomery	Huber	11	0.026	0.005
Montgomery	Lane	24	0.039	0.013
Prairie Ridge	Harvey	6	0.106	0.060
Prairie Ridge	Tombstone	24	0.052	0.017
