# Supplementary material for: Population connectivity in voles (Microtus sp.) as a gauge for tall grass prairie restoration in midwestern North America
Source: PLoS One. 2021 Dec 9;16(12):e0260344. doi: 10.1371/journal.pone.0260344 (PMC8659414; doi:10.1371/journal.pone.0260344)
Supplement: S5 Table — Comparison of population genetic and life history parameters for two vole and three bird species. (PDF) [file pone.0260344.s011.pdf]

S5 Table: Population genetic parameters for five prairie species

S5 Table. Comparison of population genetic and life history parameters for five prairie species in Illinois, including two voles and three birds

Parameter	MIOC	MIPE	PHCO	TYCU	COVI
Loci	15	15	19	21	11
A_R	12.5	12	5.34	5.35	8.63
H_o	0.8	0.7	0.62	0.65	0.71
F_{ST}	1%	-	100%	100%	100%
N_e	103	239	43	13	62.1
r	0.04	-	0.15	0.31	0.02
Breeding	Monogamy	Polygyny	Polygyny	Polygyny	Monogamy
Source	this study	this study		Douglas et al. 2020	

MIOC = *M. ochrogaster;* MIPE = *M. pennsylvanicus*; PHCO = *Phasianus colchicus* (common pheasant); TYCO = *Tympanuchus cupido pinnatus* (greater prairie chicken); COVI = *Colinus virginianus* (northern bobwhite quail). Loci = number of microsatellite DNA loci; A_R = average allelic richness; H_o = observed heterozygosity; F_{ST} = gene flow; N_e = effective population size; r = average relatedness.

Source

Douglas MR, Anthonysamy WJB, Mussmann SM, Davis MA, Louis W, Douglas ME. Multi-targeted management of upland game birds at the agroecosystem interface of Midwestern North America. PLos ONE Biodiversity. 2020; 15(4): e0230735. https//doi.org/10.1371/journal.pone.0230735
